# Measuring older people’s socioeconomic position: a scoping review of studies of self-rated health, health service and social care use

**DOI:** 10.1136/jech-2021-218265

**Published:** 2022-03-15

**Authors:** Gemma Frances Spiers, Jennifer E Liddle, Daniel Stow, Ben Searle, Ishbel Orla Whitehead, Andrew Kingston, Suzanne Moffatt, Fiona E Matthews, Barbara Hanratty

**Affiliations:** 1 Faculty of Medical Sciences, Newcastle University, Newcastle upon Tyne, UK; 2 Population and Health Sciences Institute, Newcastle University, Newcastle upon Tyne, UK

**Keywords:** RESEARCH DESIGN, METHODS, SOCIAL CLASS

## Abstract

**Background:**

The challenges of measuring socioeconomic position in older populations were first set out two decades ago. However, the question of how best to measure older people’s socioeconomic position remains pertinent as populations age and health inequalities widen.

**Methods:**

A scoping review aimed to identify and appraise measures of socioeconomic position used in studies of health inequalities in older populations in high-income countries. Medline, Scopus, EMBASE, HMIC and references lists of systematic reviews were searched for observational studies of socioeconomic health inequalities in adults aged 60 years and over, published between 2000 and 2020. A narrative synthesis was conducted.

**Findings:**

One-hundred and thirty-eight studies were included; 20 approaches to measuring socioeconomic position were identified. Few studies considered which pathways the chosen measures of socioeconomic position intended to capture. The validity of subjective socioeconomic position measures, and measures that assume shared income and educational capital, should be verified in older populations. Incomplete financial data risk under-representation of some older groups when missing data are socially patterned. Older study samples were largely homogeneous on measures of housing tenure, and to a lesser extent, measures of educational attainment. Measures that use only two response categories risk missing subtle differences in older people’s socioeconomic circumstances.

**Conclusion:**

Poor choice of measures of socioeconomic position risk underestimating the size of health inequalities in older populations. Choice of measures should be shaped by considerations of theory, context and response categories that detect subtle, yet important, inequalities. Further evidence is required to ascertain the validity of some measures identified in this review.

## Background

Socioeconomic status is a construct that reflects a person’s economic circumstances and their social capital relative to that of others.^1^ The concept is central to understanding health inequalities,^2 3^ where a gradient describes differences in health outcomes between the least and most advantaged.^4^ Both socioeconomic status and position are used in the health inequalities literature. However, some advocate for position, because status poorly distinguishes between economic resources (eg, income, wealth, education) and social prestige.^5^ While there is no consensus on this distinction and both continue to be used, we use the term socioeconomic position in recognition of this push for clarity.

Measuring socioeconomic position is challenging. Health inequalities are shaped by a complex interplay of material (eg, financial resources), psychosocial (eg, social and emotional support networks) and behavioural (eg, health behaviours) pathways linked to a person’s socioeconomic circumstance across the life course.^3 4 6^ Measures are wide-ranging,^7^ but not interchangeable.^8^ Some measures may be more relevant than others depending on which of these pathways, hypothetically, underpin unequal health outcomes.^4^ Given the different ways that (dis)advantage manifests, socioeconomic circumstances can be measured at the level of the individual, family, household or area. The relevance and fit of measures will also change depending on life stage, while the cumulative effect of (dis)advantage over time is also a critical consideration when choosing measures. Some measures will align to economic capital, and others to social capital, yet even these dimensions are multifaceted. As Galobardes *et al* note, no single measure captures the entire breadth of the influence exerted by a person’s socioeconomic position at each point in their life.^9^

In older populations, measuring socioeconomic position accrues additional complexity.^10^ Many measures are designed for working-age populations and lack relevance for older people.^11^ Historic differences between men and women in educational and workforce participation make some measures prone to gender bias.^12 13^ Others have noted that a majority in some older cohorts—particularly in the UK—are home owners.^10^ Home ownership is an importance source of wealth in older populations, yet this measure may identify very little variation if owning a home is widespread. Furthermore, home ownership can mask huge variations in house value, which may be a more sensitive marker of socioeconomic differences than ownership alone. Similar observations have been made for educational attainment, where the majority of older people in the UK have similar levels of education. As Grundy and Holt note, a measure of educational attainment may identify only the most advantaged. Whether these measures are sufficient to identify variation in socioeconomic circumstance is therefore questionable. To enable a judgement on this, evidence is needed about the extent to which study populations—including those beyond the UK—vary on these indicators.

Concerns have also been raised about the ease of collecting financial data from older people who may have numerous income sources, such as multiple pensions, savings and age-specific state welfare support (eg, Attendance Allowance in the UK, Medicare in the USA).^10 14^ Certainly, some evidence suggests that missing income data are more likely for households with more income sources.^15^ Thus, while challenges of collecting financial data are not specific to older groups, the complex income sources at older age is an important consideration and a potential limitation of income-related metrics.

A further consideration is the greater risk of cognitive impairment (mild or otherwise) in older groups, which may impact recall. This is a potential limitation to data collected from a number of socioeconomic measures, but perhaps more so for measures that require more information, such as income sources.

For older populations living in care homes, some measures become even more challenging to implement. Home ownership, for example, is an important source of wealth. Yet for those who have sold their assets to fund care, being classed as not a home owner potentially misrepresents that dimension of their economic circumstance. Similarly, income sources may be challenging to document for people living in care homes where some income is paid directly to the care provider.

Perhaps most critically, many measures of socioeconomic position risk overlooking economic resources accumulated over the life-course. Accrued economic capital, such as housing wealth (for home owners) and other long-term held assets, is important because (dis)advantage accumulates over time.^11^ Measures that differentiate between those with and without such accrued capital will therefore be particularly advantageous in identifying health inequalities in later life. Accumulated (dis)advantage in later life should not, however, be considered a resultant end-point for working-age inequalities, with welfare policies also shaping older people’s socioeconomic circumstance.^16^

### The need for review

The challenges of measuring socioeconomic position in older populations were first set out by Grundy and Holt.^10^ Two decades later, the question of how best to measure socioeconomic position in older populations remains pertinent. As people live longer with greater levels of disability,^17–20^ understanding and preventing health inequalities in ageing populations remains critical. Revisiting this issue is important and timely.

This work aimed to (1) identify which measures of socioeconomic position have been used in studies of older people’s health inequalities and (2) critically appraise the application of these measures in older populations.

## Methods

A scoping review was used to address the aims of this work. Scoping reviews map evidence in relation to a defined question or topic using systematic searches, criteria, selection process, data coding and synthesis.^21–23^ We outline the methods below according to the Preferred Reporting Items for Systematic Reviews and Meta-Analyses extension for Scoping Reviews Checklist.^24^

### Search strategy

A search strategy was developed, tested and refined based on two concepts: socioeconomic position and older people (see online supplemental material 1).

10.1136/jech-2021-218265.supp1 Supplementary data

Searches were conducted in Medline (OVID Medline and In-Process & Other Non-Indexed Citations), Scopus, EMBASE (OVID) and Health Management Information Consortium (OVID), on 24 September 2020, and limited to publications from 2000. We also checked the reference lists of relevant systematic reviews,^25 26^ and the publications of authors known to have carried out work on this topic.

### Review criteria

Observational studies were included if they examined a measure of socioeconomic position in relation to a health, health service use or social care use outcome in populations aged 60 years and over (table 1). Both populations living in the community or care homes were eligible. To identify new approaches to quantifying older people’s socioeconomic position, measures were not predefined for the review.

**Table 1:** Review criteria

	Inclusion	Exclusion
Population	Aged 60+ years. If sample include those aged less than 60 years, only studies presenting data separately for those aged 60+ are eligible. No limits were placed on setting/residence (eg, community dwelling, care home populations).	
Exposure	Any measure of socioeconomic status/position/circumstance, including but not limited to: education, wealth, financial/material resources, income, net wealth, assets, area deprivation, housing tenure, occupational classification. Measures do not need to be explicitly described as ‘socioeconomic’. Composite measures (ie, combining multiple indicators) are eligible. Subjective (eg, financial strain) or objective measures (eg, net wealth).	Socioeconomic status/position measured in childhood.
Outcome	Self-rated health. Any primary or secondary health service utilisation. Any social care or long-term care use utilisation including care homes with or without nursing, and community-based services such as home care or day centres. Outcomes must be examined in relation to a measure of socioeconomic position (see exposure, above)	Health service use does not include treatment, surgery or medication use.
Study design	Observational. English language studies. Published from 2000. Published in OECD-listed high-income countries.	Commentaries, literature reviews (unless relevant for reference checking)

OECD, Organisation for Economic and Co-operative Development.

Due to the wide variation in terminology used,^9^ it was not necessary for eligible studies to explicitly refer to such measures as ‘socioeconomic status' or 'position’. Rather, eligible studies must have examined socioeconomic inequalities in outcomes relating to health, health service utilisation and social care utilisation. Self-rated health was selected as an exemplar health outcome: it is one of the strongest indicators of health,^27^ consistently predicts mortality, including in older age groups,^28 29^ and has a high level of predictive power across the socioeconomic spectrum.^30 31^ Health service utilisation included any primary or secondary service utilisation (eg, general practice contacts, hospital admissions). We did not include studies that focused on individual aspects of the content of care, such as medications or surgical procedures. Social care or long-term care use utilisation included admission to or stays in care homes with or without nursing, and use of community based services such as home care or day centres.

Studies published before 2000 were excluded to ensure only contemporary measures were identified. Translation of non-English studies risked loss of meaning and accuracy in terminology used to describe socioeconomic position. Thus, studies not published in the English language were excluded. Finally, additional complexities of measuring socioeconomic position in low and middle income countries,^32^ which would require specialist searches, warranted the inclusion of studies only from Organisation for Economic and Co-operative Development (OECD)-listed high-income countries.^33^

### Study selection

Records were managed in Rayyan (https://rayyan.qcri.org), an online platform to assist screening for reviews.^34^ Titles and abstracts of records were screened for relevance by two researchers (GFS, DS, JEL, IOW and BS), with conflicts resolved through discussion. The full texts of selected records were obtained and assessed against the review criteria by one researcher (GFS), with 35% assessed by a second researcher (DS and JEL).

### Data extraction and synthesis

A data extraction form was developed and piloted using Excel, and relevant study information summarised. A narrative synthesis was conducted,^35^ where evidence was interrogated to address five questions (box 1).

Box 1 **Questions addressed in narrative synthesis**

1. What measures of socioeconomic position are used in studies with older populations?
2. What are the strengths and limitations of using each measure with older populations?
3. Are measures grounded in theory and justified for use in older populations?
4. Are older populations homogeneous on measures of educational attainment and housing tenure?
5. Were there any reports of difficulties collecting financial data?

### Quality assessment

An assessment of study quality and bias is important when making a judgement about the confidence and reliability of evidence. The purpose of this review was to identify and appraise measures of socioeconomic position used in older populations, and was therefore focused on methodology. The review did not synthesise evidence about the relationship between socioeconomic position and the specified outcomes. An assessment of study quality was therefore unnecessary to meet the review’s objectives.

## Findings

**Figure 1:**
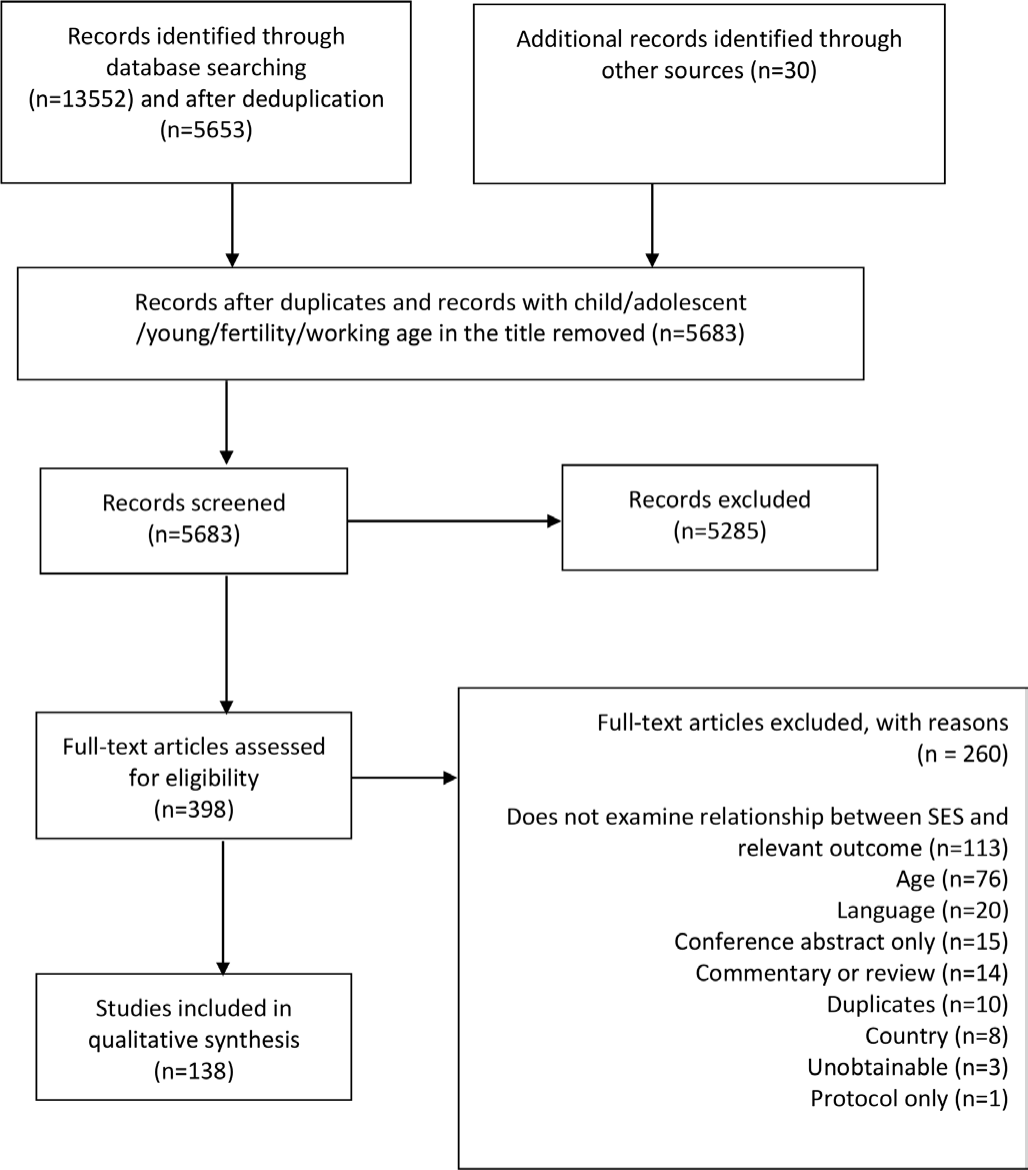
PRISMA Flowchart. SES, socioeconomic status.

One hundred and thirty-eight studies met the review criteria (figure 1 and online supplemental table S1, online supplemental materials 1 and 2). Twenty approaches to measuring socioeconomic position were identified (table 2). Fewer than half of the measures used hierarchical response categories (46.8%), from which to assess a gradient (table 3).

10.1136/jech-2021-218265.supp2 Supplementary data

**Table 2:** Measures of socioeconomic position in studies with older populations

Type of measure	No of studies using type of measure*,†
Education	86
Objective income	71
Employment/occupational classification	25
Housing tenure	22
Subjective assessment of economic circumstance	19
Area deprivation or other area level measure	16
Wealth/assets‡	14
Household material deprivation	4
Health insurance status	4
House value	3
Composite measure comprising 2+indicators	3
Car ownership	2
Geography profile of residence	2
Living arrangements§	2
Proportion of life working part time	1
Marital status§	1
Perceived access to healthcare§	1
Out of pocket payments for healthcare	1
Poverty income ratio	1
Poverty threshold status	1

*Number of studies not mutually exclusive as studies often used multiple measures.

†Figure does not represent the total number of *measures* as some studies used multiple versions of one type of measure (eg, two types of area deprivation measure).

‡Some measures were net (ie, accounted for debt and outgoings).

§Described as socioeconomic measures but their relevance to socioeconomic position unclear in publication.

**Table 3:** Response format of measures of socioeconomic position in older populations

Measure	Response format	How categorised (if applicable)
Education	3+ categories (hierarchical) 3+ categories (non-hierarchical) Count (years) Two categories	Level of education attained (years or by qualification) Quartiles/Quintiles based on years of schooling (relative) Above/below a given level
Income	Count 3+ categories (hierarchical) 3+ categories (non-hierarchical) Two categories	Quartiles/quintiles/deciles (relative and absolute) Income bands Above/below a given level
Occupational classification or employment	3+ categories (hierarchical) 3+ categories (non-hierarchical) Two categories	By occupational classification Employed/not employed
Housing tenure/home ownership	3+ categories (hierarchical) Two categories	Home owned/not owned Home owned/rented (social)/rented (private)
House value	3+ categories (hierarchical)	House value bands
(Net) Assets	3+ categories (hierarchical) Count	Total value of worth/wealth bands Quartiles/quintiles/deciles (relative)
Subjective SEP	3+ categories (hierarchical) Two categories	Rating of circumstance Yes/no
Area deprivation or other area measure	3+ categories (hierarchical) Score/proportion Index	Quartiles/quintiles (relative and absolute)
Car ownership	Count Two categories	Owned/no owned car
Insurance status	Two categories 3+ categories (hierarchical)	Whether participant meets an insurance threshold signalling low income None/public/private Has private/public insurance yes/no
Geography profile of residence	Two categories	Metropolitan/non-metropolitan Urban/rural
Living lrrangements	Two categories 3+ categories (non-hierarchical)	Alone/not alone Live with spouse/live with other/live alone
Household material deprivation	3+ categories (hierarchical) Two categories	0, 1 or 2+basic items lacked <3 or 3+household items unable to afford Good/bad, based on split of a household conditions index
Proportion of life working part time	Proportion	NA
Marital status	Two categories	Married/other
Perceived access to healthcare	Score (0–1)	NA
Out of pocket payments for healthcare	Two categories	Yes/no
Poverty income ratio	3+ categories (hierarchical)	Bands of poverty-income ratio
Poverty threshold status	Two categories	Above or below a given poverty threshold

N/A, not available; SEP, socioeconomic position.

In this section, we present findings according to the data interrogation questions that guided the synthesis (box 1).

### What are the strengths and limitations of measures of socioeconomic position in older populations?

Online supplemental table S2 summarises the strengths and limitations of applying each identified measure to older populations. This builds on the challenges set out previously,^10^ and highlights additional considerations. From this appraisal, we summarise three issues that have not been previously explored in relation to measuring socioeconomic position in older populations and which warrant further scrutiny.

#### Subjective socioeconomic position

These measures reflected older people’s self-assessed satisfaction with economic circumstance, perceived adequacy of income or economic resources and perceived financial security, strain and problems. Applied to older populations, a subjective measure could overcome the challenges of collecting financial data that may be sensitive and/or wide-ranging. However, older people tend to rate their economic situation better than it objectively appears.^36^ Subjective assessments of economic circumstance may also be compounded by the health of the individual. Furthermore, subjective ratings depend on whom people use as a reference for comparison, which could change over time and between circumstances. Thus, while a subjective assessment may be an attractive option for measuring socioeconomic position in older populations, evidence is needed to ascertain the validity of this approach.

#### Incorporating others’ income in measures

Income was measured at the level of the individual, family or household. Measures of household or family income may reflect two circumstances: cohabiting older couples and older people living with younger family members. In the latter circumstance, a measure of household or family income assumes that an older person benefits from this shared resource, thus enhancing their position of advantage. However, the reverse is also possible. Other household and family members may benefit from the income of older family members, thus potentially depleting this resource and lowering their level of advantage. Income sharing within households is complex, influenced by who is the primary earner, family type, power dynamics and consumption levels.^37–40^ The extent to which an older person may or may not benefit, if at all, from others’ incomes may therefore be highly variable.

#### Household educational attainment

While most studies measured the older person’s attainment, two studies measured the highest educational attainment within the household. Study authors argued that older members of the household could benefit from potentially greater levels of education of other household members. This approach may overcome the challenge of potential homogeneity in older people’s educational attainment. However, it is unclear to what extent the benefits of educational attainment: are shared within households and families; relate to material circumstance; and, represents a valid approach to measuring older people’s socioeconomic position.

### Are measures grounded in theory and justified for use in older populations?

Typically, studies did not explain which pathway to inequality (ie, behavioural, materialist and psychosocial) their chosen measure intended to capture within the older population. Pragmatism drove some choices, where proxy measures were used in the absence of other data. Study authors seldom reflected on the limitations of chosen socioeconomic position measures within older populations.

### Are older populations homogeneous on measures of educational attainment and housing tenure?

Online supplemental table S3 summarises the spread of study samples by categories of educational attainment and home ownership, where these data were reported in studies using these measures. A small number of studies demonstrated homogeneous levels of educational attainment within their older population, where over 70% of the sample was classified in the lowest attainment category. Where these data were reported by sex, the proportion in the lowest educational attainment category was usually higher for women.

On measures of housing tenure, older populations in most studies were often home owners, who typically comprised more than 70% of study samples (online supplemental table S3). This pattern may reflect current trends in home ownership, or it could be a product of selection biases in study samples.

### Did studies report difficulties collecting financial data?

There was some evidence that financial data (income and assets) were difficult to collect, with reports of missing data. There was also some evidence that missing data were socially patterned. Non-report of income data was most likely for home owners (one study), those with lower educational attainment (one study), older female participants (one study), those over 75 years (one study) and those in poor health (one study).

## Discussion

Previous work has highlighted the challenges of measuring socioeconomic position in older populations.^10^ This scoping review has updated and expanded this work, providing a comprehensive picture of the approaches that have been used in contemporary studies of health inequalities. We now consider what factors should drive the choice of measure and where further evidence is needed about measuring older people’s socioeconomic position.

### What should be considered when choosing measures of socioeconomic position in studies of health inequalities in older populations?

While different approaches to measuring older people’s socioeconomic position have merits and drawbacks, choice of measures should be driven by three broad considerations.

- (1) As previously advocated,^3 4 6 8^ measures of socioeconomic position should be chosen based on what aspect of older people’s socioeconomic circumstance is thought to underlie unequal health outcomes. Where material conditions are proposed to underpin unequal outcomes, measures that combine income, home ownership or other assets may best capture accumulated economic capital in later life. Some studies did indeed use this approach, but it was not common. The most common measure was educational attainment. Yet early life education may be a weak indicator of later life resources, especially where social mobility has played a more important role in boosting employment opportunities.
- (2) Measures should be chosen with consideration of how macroeconomic and policy contexts shape the socioeconomic profile of the older population studied, including how these contexts change over time. Education and housing continue to be popular measures, yet the extent to which an older population varies on these indicators is shaped by the public policies to which they are exposed over the life course. Similarly, macroeconomic factors influence subjective assessments of economic circumstance.^41 42^ These influences, which may differ by country, means that the ‘fit’ of a measure to older populations will vary across contexts and make cross-country comparisons challenging.
- Changes in policy contexts *ov*er time should also be considered when choosing measures. For example, trends in home ownership are declining among 18–34 years olds across Europe.^43^ Home ownership as a measure of socioeconomic position may, therefore, capture greater heterogeneity for future older cohorts if such trends in ownership continue. Similarly, educational attainment may become more varied for later (and future) older cohorts in countries where access to postschool education is increasing.^44^
- (3) Measures should maximise the detection of subtle variations in older people’s socioeconomic circumstance. Measures often used two response categories, which may be due to limitations in sample size, or minimal variation on the measure. Even so, in the oldest old, the gap between the most and least advantaged may be smaller due to premature mortality in lower socioeconomic groups.^45^ A two-category measure will be too blunt to detect these subtle, yet important, differences. While some binary measures capture key aspects of material resource (eg, home ownership), used in isolation such measures may be unhelpful.
- Inevitably, choice of measures is often pragmatic and based on available data. This presents an important consideration for future longitudinal cohort studies of older populations, particularly those with long-term prospective follow-up periods. Data collected on a range of socioeconomic indicators will enable researchers to (1) choose those most theoretically relevant to the study question and population, (2) respond to changes in populations and contexts over time and (3) select multiple measures to capture different dimensions of (dis)advantage over the life-course.

### Where is more evidence needed to ascertain the validity of approaches to measuring older people’s socioeconomic position?

Subjective assessments of socioeconomic position may be compounded by health status and a tendency for older people to rate their economic circumstances favourably.^36^ Further research could explore the validity of this approach, including how assessments vary over time and between contexts. Measures that include others’ educational attainment and income require verification about whether shared capital (from income or education) equates to greater or lower levels of advantage for the older person, and the validity of these approaches with older populations.

Earlier concerns about missing financial data were also confirmed in a small number of studies.^46–54^ While there is evidence that missing income data is socially patterned in studies not specific to older groups,^55 56^ there is less clarity about the patterning specifically at older ages. Further research could explore whether it is the most or least advantaged older people who are most likely to be under-represented on these measures. This is important given that participation in cohort studies is already biased towards more advantaged populations.^57^

Finally, the changing nature of economic factors shaping a person’s socioeconomic circumstance means that the best measurement approaches will differ for future older cohorts. Falling rates of home ownership,^58^ projected lower retirement incomes^59^ and women’s increased labour force participation,^60^ necessitate regular assessment of the fit of measures in older populations. A related point is that the limitations of some socioeconomic position measures in older groups may also become relevant to younger groups as populations, policies and the economy evolve. The extent to which the challenges of socioeconomic position measures outlined in this paper remain specific to, or extend beyond, older populations in future is important to monitor.

### Strengths and limitations

Systematic scoping methods have provided a comprehensive up-to-date picture of the socioeconomic position measures used in contemporary studies with in older populations. We have expanded earlier appraisals of existing measures, appraised new approaches and identified gaps where evidence is needed to ascertain the validity of measures.

We focused our review on studies examining inequalities in health and social care use, and self-rated health. Health outcomes are wide ranging and it would not have been possible to include studies of all such outcomes here. This is an important limitation; studies using other health outcomes may have implemented socioeconomic measures not identified here. Thus, we do not claim that our review offers a complete picture of measures of socioeconomic position in older populations. Self-rated health was chosen as the exemplar health outcome because it is one of the most common and strongest measures of health.^27^ Inevitably, this measure is also subject to variations between countries, age, gender, ethnicity and socioeconomic position.^31 61 62^ However, these limitations have little impact on the work reported here as we did not make comparisons of self-rated health between these groups.

No studies were identified that explicitly used ethnicity as a proxy for socioeconomic position. Ethnicity is sometimes used as a measure of socioeconomic position in US studies, and the two are often conflated.^63^ That is, both are related to each other but have independent effects on health outcomes. The absence of studies using ethnicity as a measure of socioeconomic position is not a major shortcoming, as the limitations of this approach have been highlighted previously.^63^

## Conclusion

Choosing measures of older people’s socioeconomic position should be shaped by considerations of theory, context and opportunity to assess a gradient. Some measures require evidence to ascertain their validity. Measures should be reviewed regularly to assess fit for purpose in older populations, as socioeconomic profiles, economic and public policies change over time.

**What is already known on this topic**

- Measuring socioeconomic position in older populations is challenging.

**What this study adds**

- Choosing measures of older people’s socioeconomic position should be shaped by theory, context and opportunity to assess a gradient.
- Some measures require evidence to ascertain their validity in older populations.

**How this study might affect research, policy and/or practice**

- Measures should be reviewed regularly as socioeconomic profiles, economic and public policies change over time

## Data Availability

Data sharing not applicable as no datasets generated and/or analysed for this study. No datasets were generated and/or analysed for this study.

## References

[1] Cutler D , Lleras-Muney A , Vogl T . Socioeconomic status and health: dimensions and mechanisms. NBER Working Paper, 14333. Cambridge, USA: National Bureau of Economic Research, 2008.

[2] Adler NE , Boyce T , Chesney MA , et al . Socioeconomic status and health. The challenge of the gradient. Am Psychol 1994;49:15–24. 10.1037//0003-066x.49.1.15 8122813

[3] Adler NE , Ostrove JM . Socioeconomic status and health: what we know and what we don't. Ann N Y Acad Sci 1999;896:3–15. 10.1111/j.1749-6632.1999.tb08101.x 10681884

[4] Bartley M . Health inequality: an introduction to concepts, theories and methods. 2nd Edition. Polity Press, 2017.

[5] Krieger N , Williams DR , Moss NE . Measuring social class in US public health research: concepts, methodologies, and guidelines. Annu Rev Public Health 1997;18:341–78. 10.1146/annurev.publhealth.18.1.341 9143723

[6] van Oort FVA , van Lenthe FJ , Mackenbach JP . Material, psychosocial, and behavioural factors in the explanation of educational inequalities in mortality in the Netherlands. J Epidemiol Community Health 2005;59:214–20. 10.1136/jech.2003.016493 15710599 PMC1733027

[7] Galobardes B , Shaw M , Lawlor DA , et al . Indicators of socioeconomic position (Part 2). J Epidemiol Community Health 2006;60:95–101. 10.1136/jech.2004.028092 16415256 PMC2566160

[8] Braveman PA , Cubbin C , Egerter S , et al . Socioeconomic status in health research: one size does not fit all. JAMA 2005;294:2879–88. 10.1001/jama.294.22.2879 16352796

[9] Galobardes B , Shaw M , Lawlor DA , et al . Indicators of socioeconomic position (Part 1). J Epidemiol Community Health 2006;60:7–12. 10.1136/jech.2004.023531 PMC2465546 16361448

[10] Grundy E , Holt G . The socioeconomic status of older adults: how should we measure it in studies of health inequalities? J Epidemiol Community Health 2001;55:895–904. 10.1136/jech.55.12.895 11707484 PMC1731799

[11] Crystal S , Shea D . Cumulative advantage, cumulative disadvantage, and inequality among elderly people. Gerontologist 1990;30:437–43. 10.1093/geront/30.4.437 2394380

[12] Scharf T , Shaw C . Inequalities in later life: centre for ageing better, 2017.

[13] Roantree B , Vira K . The rise and rise of women’s employment in the UK. IFS Briefing Note BN234: Institute for Fiscal Studies, 2018.

[14] Gornick JC , Sierminska E , Smeeding TM . The income and wealth packages of older women in cross-national perspective. J Gerontol B Psychol Sci Soc Sci 2009;64:402–14. 10.1093/geronb/gbn045 19208754

[15] Frick JR , Grabka MM . Missing income data in the German SOEP: incidence, imputation and its impact on the income distribution, SOEP survey papers, no. 225. Berlin: Deutsches Institut für Wirtschaftsforschung (DIW), 2014.

[16] Higgs P , Formosa M . The changing significance of social class in later life. In: Formosa M , Higgs P , eds. Social class in later life power, identity and lifestyle. Great Britain: Policy Press, 2015.

[17] Jagger C , Collerton JC , Davies K , et al . Capability and dependency in the Newcastle 85+ cohort study. projections of future care needs. BMC Geriatr 2011;11:21. 10.1186/1471-2318-11-21 21542901 PMC3097155

[18] Kingston A , Comas-Herrera A , Jagger C , et al . Forecasting the care needs of the older population in England over the next 20 years: estimates from the population ageing and care simulation (PACSim) modelling study. Lancet Public Health 2018;3:e447–55. 10.1016/S2468-2667(18)30118-X 30174210 PMC6123499

[19] Kingston A , Robinson L , Booth H , et al . Projections of multi-morbidity in the older population in England to 2035: estimates from the population ageing and care simulation (PACSim) model. Age Ageing 2018;47:374–80. 10.1093/ageing/afx201 29370339 PMC5920286

[20] Wittenberg R , Hu B . Projections of demand for and costs of social care for older people and younger adults in England 2015-2035: personal social services research unit, 2015.

[21] Arksey H , O'Malley L . Scoping studies: towards a methodological framework. Int J Soc Res Methodol 2005;8:19–32. 10.1080/1364557032000119616

[22] Colquhoun HL , Levac D , O'Brien KK , et al . Scoping reviews: time for clarity in definition, methods, and reporting. J Clin Epidemiol 2014;67:1291–4. 10.1016/j.jclinepi.2014.03.013 25034198

[23] Levac D , Colquhoun H , O'Brien KK . Scoping studies: advancing the methodology. Implement Sci 2010;5:69. 10.1186/1748-5908-5-69 20854677 PMC2954944

[24] Tricco AC , Lillie E , Zarin W , et al . PRISMA extension for scoping reviews (PRISMA-ScR): checklist and explanation. Ann Intern Med 2018;169:467–73. 10.7326/M18-0850 30178033

[25] Almeida APSC , Nunes BP , Duro SMS , et al . Socioeconomic determinants of access to health services among older adults: a systematic review. Rev Saude Publica 2017;51:50. 10.1590/S1518-8787.2017051006661 28513761 PMC5779074

[26] Read S , Grundy E , Foverskov E . Socio-Economic position and subjective health and well-being among older people in Europe: a systematic narrative review. Aging Ment Health 2016;20:529–42. 10.1080/13607863.2015.1023766 25806655 PMC4784497

[27] Fayers PM , Sprangers MAG . Understanding self-rated health. Lancet 2002;359:187–8. 10.1016/S0140-6736(02)07466-4 11812551

[28] Falk H , Skoog I , Johansson L , et al . Self-Rated health and its association with mortality in older adults in China, India and Latin America-a 10/66 dementia research Group study. Age Ageing 2017;46:932–9. 10.1093/ageing/afx126 28985329 PMC5860352

[29] DeSalvo KB , Bloser N , Reynolds K , et al . Mortality prediction with a single General self-rated health question. A meta-analysis. J Gen Intern Med 2006;21:267–75. 10.1111/j.1525-1497.2005.00291.x 16336622 PMC1828094

[30] Burström B , Fredlund P . Self rated health: is it as good a predictor of subsequent mortality among adults in lower as well as in higher social classes? J Epidemiol Community Health 2001;55:836–40. 10.1136/jech.55.11.836 11604441 PMC1763304

[31] Dowd JB , Zajacova A . Does the predictive power of self-rated health for subsequent mortality risk vary by socioeconomic status in the us? Int J Epidemiol 2007;36:1214–21. 10.1093/ije/dym214 17971388

[32] Fotso J-C , Kuate-Defo B . Measuring socioeconomic status in health research in developing countries: should we be focusing on households, communities or both? Soc Indic Res 2005;72:189–237. 10.1007/s11205-004-5579-8

[33] World Bank Group . Fact sheet: OECD high-income: world bank, 2019.

[34] Ouzzani M , Hammady H , Fedorowicz Z , et al . Rayyan-a web and mobile APP for systematic reviews. Syst Rev 2016;5:210. 10.1186/s13643-016-0384-4 27919275 PMC5139140

[35] Higgins JPT , Green S . Cochrane Handbook for systematic reviews of interventions version 5.1.0, 2011. Available: http://handbook.cochrane.org/front_page.htm [Accessed 19 Jan 2017].

[36] Price D . Measuring the poverty of older people: a critical review. London: Economic & Social Research Council, Institute of Genrontology, 2008.

[37] Pepin JR . Beliefs about money in families: balancing unity, autonomy, and gender equality. J Marriage Fam 2019;81:361–79. 10.1111/jomf.12554

[38] Eickmeyer KJ , Manning WD , Brown SL . What’s Mine Is Ours? Income Pooling in American Families. J Marriage Fam 2019;81:968–78. 10.1111/jomf.12565

[39] Bonke J . Pooling of income and sharing of consumption within households. Rev Econ Househ 2015;13:73–93. 10.1007/s11150-013-9184-y

[40] Bonke J , Uldall-Poulsen H . Income pooling within families – survey evidence of Denmark. globalization, society and welfare. Working paper 05:2005: the Danish National Institute of social research, 2005.

[41] Glei DA , Goldman N , Weinstein M . Perception has its own reality: subjective versus objective measures of economic distress. Popul Dev Rev 2018;44:695–722. 10.1111/padr.12183 30828111 PMC6395043

[42] Vauclair C-M , Marques S , Lima ML , et al . Subjective social status of older people across countries: the role of modernization and employment. J Gerontol B Psychol Sci Soc Sci 2015;70:650–60. 10.1093/geronb/gbu074 24942971

[43] Arundel R , Doling J . The end of mass homeownership? changes in labour markets and housing tenure opportunities across Europe. J Hous Built Environ 2017;32:649–72. 10.1007/s10901-017-9551-8 29323352 PMC5744615

[44] United Nations Educational SaCO . Towards universal access to higher education: international trends, 2020.

[45] Bowling A . Socioeconomic differentials in mortality among older people. J Epidemiol Community Health 2004;58:438–40. 10.1136/jech.2003.017582 15143105 PMC1732791

[46] Hancock R , Arthur A , Jagger C , et al . The effect of older people's economic resources on care home entry under the United Kingdom's long-term care financing system. J Gerontol B Psychol Sci Soc Sci 2002;57:S285–93. 10.1093/geronb/57.5.s285 12198108

[47] Angel RJ , Frisco M , Angel JL , et al . Financial strain and health among elderly Mexican-origin individuals. J Health Soc Behav 2003;44:536–51. 15038148

[48] Robert SA , Cherepanov D , Palta M , et al . Socioeconomic status and age variations in health-related quality of life: results from the National health measurement study. J Gerontol B Psychol Sci Soc Sci 2009;64:378–89. 10.1093/geronb/gbp012 19307286 PMC2670253

[49] Robert SA , Lee KY . Explaining race differences in health among older adults: the contribution of community socioeconomic context. Research on Aging 2002;24:654–83. 10.1177/016402702237186

[50] Allan DE , Funk LM , Reid RC , et al . Exploring the influence of income and geography on access to services for older adults in British Columbia: a multivariate analysis using the Canadian community health survey (cycle 3.1). Can J Aging 2011;30:69–82. 10.1017/S0714980810000760 21366934

[51] Fernandez-Martinez B , Prieto-Flores M-E , Forjaz MJ , et al . Self-Perceived health status in older adults: regional and sociodemographic inequalities in Spain. Rev Saude Publica 2012;46:310–9. 10.1590/s0034-89102012000200013 22437859

[52] Freedman VA , Rogowski J , Wickstrom SL , et al . Socioeconomic disparities in the use of home health services in a Medicare managed care population. Health Serv Res 2004;39:1277–97. 10.1111/j.1475-6773.2004.00290.x 15333109 PMC1361070

[53] Ornstein KA , Garrido MM , Bollens-Lund E , et al . The association between income and incident Homebound status among older Medicare beneficiaries. J Am Geriatr Soc 2020;68:2594–601. 10.1111/jgs.16715 32776512 PMC7722026

[54] Huijts T , Eikemo TA , Skalická V . Income-Related health inequalities in the Nordic countries: examining the role of education, occupational class, and age. Soc Sci Med 2010;71:1964–72. 10.1016/j.socscimed.2010.09.021 20943303

[55] Park HA . Rate of missing socioeconomic factors in the 4th KNHANES. Korean J Fam Med 2012;33:406–9. 10.4082/kjfm.2012.33.6.406 23267427 PMC3526724

[56] Yan T , Curtin RT , Jans ME . Trends in income nonresponse over two decades. Journal of Official Statistics 2010;26:145–64.

[57] Galea S , Tracy M . Participation rates in epidemiologic studies. Ann Epidemiol 2007;17:643–53. 10.1016/j.annepidem.2007.03.013 17553702

[58] UK Parliament . Home ownership and renting: demographics, 2017. Available: http://researchbriefings.parliament.uk/ResearchBriefing/Summary/CBP-7706#fullreport [Accessed 9 Nov 2017].

[59] Resolution Foundation,, Intergenerational Commission . As good as it gets? the adequacy of retirement income for current and future generations of pensioners: resolution Foundation, 2017.

[60] Office for National Statistics . Women in the labour market: 2013. Office for National Statistics, 2013.

[61] Bardage C , Pluijm SMF , Pedersen NL , et al . Self-Rated health among older adults: a cross-national comparison. Eur J Ageing 2005;2:149–58. 10.1007/s10433-005-0032-7 28794727 PMC5547684

[62] Young H , Grundy E , O'Reilly D , et al . Self-Rated health and mortality in the UK: results from the first comparative analysis of the England and Wales, Scotland, and Northern Ireland longitudinal studies. Popul Trends 2010;139:11–36. 10.1057/pt.2010.3 20379276

[63] LaVeist TA . Disentangling race and socioeconomic status: a key to understanding health inequalities. J Urban Health 2005;82:iii26–34. 10.1093/jurban/jti061 15933328 PMC3455905

