# Correction: *Measuring older people’s socioeconomic position: a scoping review of studies of self-rated health, health service and social care use*

**DOI:** 10.1136/jech-2021-218265corr1

**Published:** 2026-04-08

Spiers GF, Liddle JE, Stow D, *et al.* Measuring older people’s socioeconomic position: a scoping review of studies of self-rated health, health service and social care use. *J Epidemiol Community Health* 2022;76:572–579.

The second author’s name is currently listed incorrectly as Liddle JE. The correct citation should be Liddle J (Jennifer Liddle).

