# Supplementary material for: Measuring older people’s socioeconomic position: a scoping review of studies of self-rated health, health service and social care use
Source: J Epidemiol Community Health. 2022 Mar 15;76(6):572–9. doi: 10.1136/jech-2021-218265 (PMC9118079; doi:10.1136/jech-2021-218265)
Supplement: Supplementary data [file jech-2021-218265supp001.pdf]

SUPPLEMENTARY MATERIALS

Search strategy (Medline)

<u>Medline</u> Ovid MEDLINE(R) and In-Process & Other Non-Indexed Citations 1946 to September 22, 2020

Date searched: 24/09/2020

1	wealth.m_titl.
2	income.m_titl.
3	economic.m_titl.
4	financial.m_titl.
5	socio-economic.m_titl.
6	socioeconomic.m_titl.
7	"asset*".m_titl.
8	resources.m_titl.
9	"inequalit*".m_titl. 9988
10	1 or 2 or 3 or 4 or 5 or 6 or 7 or 8 or 9
11	"old*".m_titl.
12	limit 11 to yr="2000 -Current"
13	elderly.m_titl.
14	limit 13 to yr="2000 -Current"
15	retire.m_titl.
16	limit 15 to yr="2000 -Current"
17	12 or 14 or 16
18	10 and 17
19	age.m_titl.
20	limit 19 to yr="2000 -Current"
21	12 or 14 or 16 or 20
22	10 and 21

Table S1. Overview of studies (shaded cell indicates measure used)

STUDY AUTHOR	DATE	Outcome group	Lower age threshold	COUNTRY	Education	(Net) Assets	Housing tenure	House value	Housing conditions	Occupational class/employment	Income	Area deprivation or other area level	Subjective SES	Health insurance status	Car ownership	% of life working part time	Geography of residence	Marital status	Composite measure	Living arrangements	Out of pocket healthcare payments	Poverty income ratio	Poverty threshold status	Household material deprivation	Access to healthcare
Adjei	2017	Self-rated health	65	Multiple	■		■			■					■										
Ahn	2012	Self-rated health	60	US	■						■		■												
Aida	2011	Self-rated health	65	Japan	■						■														
Allen	2011	Health service use	65	Canada	■						■														
Allin	2009	Health service use	65	Multiple	■	■					■														
Alwan	2007	Health service use and self-rated health	65	UK								■													
Ament	2012	Self-rated health	70	Netherlands	■								■												
Ancona	2007	Health service use	75	Italy							■														
Angel	2003	Self-rated health	64	US	■						■		■												
Aschan-Leygonie	2013	Health service use	65	France								■													
Assari	2020	Self-rated health	65	USA	■								■												
Auchincloss	2001	Health service use	65	US	■						■														
Bambra	2010	Self-rated health	60	Multiple	■																				
Breeze	2001	Self-rated health	67	UK			■			■					■										
Cain	2017	Self-rated health	65	USA	■						■														
Cohen	2013	Health service use	65	Canada							■														
Connelly	2010	Self-rated health	65	Northern Ireland	■		■	■		■															
Dalstra	2006	Self-rated health	60	Multiple	■		■				■														
Elovainio	2000	Self-rated health	75	Finland									■												
Enroth	2013	Self-rated health	90	US	■					■															
Enroth	2019	Self-rated health	75	Multiple	■																				
Evans	2008	Self-rated health	60	USA							■														
Fernandez-Martinez	2012	Self-rated health	60	Spain									■												
Fernandez-Mayorales	2000	Health service use	65	Spain	■					■															
Fors	2015	Self-rated health	77	Sweden	■																				
Francois	2011	Health service use	65	Belgium	■		■				■														
Franse [a]	2017	Self-rated health	70	Netherlands	■							■													
Freedman	2004	Health service use	65	US	■	■	■				■														
Fukuda [a]	2015	Health service use	65	Japan						■	■						■	■							

STUDY AUTHOR	DATE	Outcome group	Lower age threshold	COUNTRY	Education	(Net) Assets	Housing tenure	House value	Housing conditions	Occupational class/employment	Income	Area deprivation or other area level	Subjective SES	Health insurance status	Car ownership	% of life working part time	Geography of residence	Marital status	Composite measure	Living arrangements	Out of pocket healthcare payments	Poverty income ratio	Poverty threshold status	Household material deprivation	Access to healthcare
Gill	2004	Health service use	77	Australia								■													
Giron	2012	Self-rated health	65	Spain	■					■	■														
Gomez-Baya [a]	2020	Self-rated health	65	Spain	■						■	■													
Grau	2001	Self-rated health	65	USA	■						■														
Grundy	2007	Social care use	65	Multiple			■																		
Grundy	2003	Self-rated health	65	UK	■		■				■														
Hamada	2019	Health service use	75	Japan							■														
Hancock	2002	Social care use	75	UK			■				■														
Hardy	2011	Health service use	66	USA								■													
Himes	2000	Social care use	70	USA and Germany							■														
Hoebel[a]	2017	Self-rated health	65	Germany															■						
Hoeck	2013	Health service use	65	Belgium	■		■																		
Honjo [a]	2006	Self-rated health	61	Japan	■						■														
Howard	2006	Self-rated health	65	USA	■						■														
Huang	2018	Health service use	65	US								■													
Huijts	2010	Self-rated health	63	Denmark, Finland	■					■	■														
Ichida	2009	Self-rated health	65	Japan	■		■				■	■													
Ilinca	2017	Social care use	60	Multiple							■														
Illoabuchi	2014	Health service use	65	US	■																				
Jenkins	2002	Social care use	65	USA							■														
Jenkins	2020	Social care use	65	USA															■						
Jiang	2020	Health service use	75	Japan							■														
Jyvakorpi	2018	Self-rated health	82	Finland									■												
Kim	2012	Health service use	65	Korea	■					■	■	■													
Kim	2011	Self-rated health	65	Korea	■					■	■												■		
Kim	2017	Self-rated health	65	Korea	■					■				■											
Kim	2008	Self-rated health	65	Korea		■																			
Kiuchi	2018	Health service use	65	Japan							■														
Kiula	2007	Self-rated health	65	US	■						■														
Knurowski	2005	Self-rated health	65	Poland	■		■			■	■														
Lakdawalla	2003	Social care use	70	USA	■						■														
Lasheras	2001	Self-rated health	65	Spain	■																				
Law	2017	Health service use	66	Canada							■														
Lee	2020	Health service use	65	US	■						■														
Li	2008	Self-rated health	60	US	■						■	■													
Lima-Costa	2012	Self-rated health	60	England							■														

STUDY AUTHOR	DATE	Outcome group	Lower age threshold	COUNTRY	Education	(Net) Assets	Housing tenure	House value	Housing conditions	Occupational class/employment	Income	Area deprivation or other area level	Subjective SES	Health insurance status	Car ownership	% of life working part time	Geography of residence	Marital status	Composite measure	Living arrangements	Out of pocket healthcare payments	Poverty income ratio	Poverty threshold status	Household material deprivation	Access to healthcare
Lopez-de-Andres	2018	Health service use and social care use	65	Spain	■						■														
Low	2009	Self-rated health	60	Canada	■	■																			
Luchetti	2009	Health service use	70	Italy	■								■												
Lum	2004	Self-rated health	70	USA	■	■																			
Lupi-Pegurier	2011	Health service use	60	France	■						■														
Maniecka-Bryła	2011	Self-rated health	65	Poland	■																				
Martinkainen	2009	Social care use	65	Finland			■				■														
Martinkainen	2008	Social care use	65	Finland	■		■			■	■														
Mather	2014	Self-rated health	65	Australia	■						■	■													
McCann	2011	Social care use	65	Northern Ireland			■	■																	
McFadden [a]	2008	Self-rated health	60	UK						■															
McMunn [a]	2009	Self-rated health	60	UK		■																			
Merlo	2003	Health service use	60	Sweden							■														
Mishra	2004	Health service use	70	Australia															■						
Muckenhuber	2014	Self-rated health	70	Austria	■																				
Munford	2017	Health service use	65	UK	■															■					
Murata	2019	Health service use and social care use	75	Japan							■														
Nicklett	2011	Self-rated health	65	US	■	■					■														
Niefield	2005	Health service use	65	US			■						■								■				
Nieman	2014	Health service use	70	US	■						■											■			
Nihtila	2007	Social care use	65	Finland	■		■			■	■				■										
Nihtila	2008	Social care use	65	Finland	■		■			■	■														
Nummela	2007	Self-rated health	62	Finland	■						■		■												
Orfila	2000	Self-rated health	65	Spain	■					■															
Ornstein	2020	Social care use	65	US						■															
Otaki	2017	Self-rated health	70	Japan									■												
Park	2020	Social care use	65	US	■		■				■														
Park	2014	Health service use and self-rated health	65	South Korea	■						■														
Park	2009	Self-rated health	65	Korea		■					■														
Patel	2007	Health service use	60	UK						■															
Pirani	2012	Self-rated health	65	Italy	■				■				■												
Pirani	2012	Self-rated health	65	Italy	■				■				■												

STUDY AUTHOR	DATE	Outcome group	Lower age threshold	COUNTRY	Education	(Net) Assets	Housing tenure	House value	Housing conditions	Occupational class/employment	Income	Area deprivation or other area level	Subjective SES	Health insurance status	Car ownership	% of life working part time	Geography of residence	Marital status	Composite measure	Living arrangements	Out of pocket healthcare payments	Poverty income ratio	Poverty threshold status	Household material deprivation	Access to healthcare
Piumatti	2017	Self-rated health	65	Italy									■												
Prajsner	2015	Health service use	65	Poland	■						■														
Prajsner	2016	Health service use	65	Poland	■						■														
Ramsay	2018	Self-rated health	71	UK								■													
Rathore	2006	Health service use	65	US								■													
Reyes-Ortiz	2010	Health service use	75	US	■						■		■	■											
Robert	2009	Self-rated health	65	US	■	■					■														
Robert	2002	Self-rated health	60	US	■	■					■	■													
Roberts[a]	2001	Self-rated health	60	US	■	■					■														
Rodrigues	2017	Social care use	60	Multiple		■					■														
Roe-Prior	2007	Health service use	65	USA	■					■	■														
Rostad	2009	Self-rated health	75	Norway	■					■															
Rueda	2012	Self-rated health	65	Spain	■							■													
Rueda	2008	Self-rated health	65	Spain	■						■														
Rueda	2009	Self-rated health	65	Spain	■																			■	
Schmidt	2017	Social care use	60	Austria							■														
Schmitz	2017	Self-rated health	60	Germany	■		■						■											■	
Shea	2003	Social care use	75	Multiple	■																				
Shebehe	2018	Health service use	65	Sweden								■													
Sheifer	2000	Health service use	65	USA								■													
Sherman	2012	Self-rated health	75	Sweden	■																				
Siciliani[a]	2009	Health service use	65	Multiple	■						■														
Sigurdardottir	2019	Self-rated health	65	Iceland	■								■				■			■					■
Stone	2015	Self-rated health	64	UK				■		■						■									
Sulander	2012	Self-rated health	75	Finland	■								■												
Sulander	2009	Self-rated health	65	Finland	■																				
Suominen-Taipale	2004	Health service use	65	Multiple	■																				
Tigani	2012	Self-rated health	100	Greece	■						■		■												
Tomiak	2000	Social care use	65	Canada	■						■														
Torssander	2016	Health service use	60	Sweden	■																				
Trachte	2016	Self-rated health	65	Germany	■						■														
Van den Bosch	2013	Social care use	65	Belgium										■											
van Groenou[a]	2006	Social care use	65	Multiple	■		■			■															
van Oorti	2003	Self-rated health	65	Belgium							■														
von dem Knesebeck	2003	Self-rated health	60	Multiple	■	■	■			■	■														

STUDY AUTHOR	DATE	Outcome group	Lower age threshold	COUNTRY	Education	(Net) Assets	Housing tenure	House value	Housing conditions	Occupational class/employment	Income	Area deprivation or other area level	Subjective SES	Health insurance status	Car ownership	% of life working part time	Geography of residence	Marital status	Composite measure	Living arrangements	Out of pocket healthcare payments	Poverty income ratio	Poverty threshold status	Household material deprivation	Access to healthcare
von dem Knesebeck	2015	Self-rated health	65	Germany	■					■	■														
Wachelder	2017	Health service use	65	Netherlands								■													
Walker	2006	Health service use	60	Australia							■														
Wang	2014	Self-rated health	65	Japan	■						■														
Wastesson	2014	Health service use	77	Sweden	■					■	■														
Williams	2008	Health service use	65	US	■	■																			

[a] Study population included those aged <60 years, but data presented separately for 60+ population

Table S2 Strengths and limitations of measures of socioeconomic position in older populations

MEASURE	Strengths and limitations
**Education**	*Strengths*<br>• Data are easy to obtain, often available in cohort datasets.<br>• Potentially comparable between countries.
	Limitations<br>• Level of educational attainment can be homogenous for older populations.<br>• Not necessarily a key driver of later life material advantage: in some countries such as the UK, labour market opportunities and conditions in 20th century may have played a more significant role than early life education in shaping employment and later life material resources.<br>• Gender bias may exist.<br>• Highest household/ family educational attainment may overcome homogeneity of this measure, but it is unclear to what extent older people benefit from the education of younger household members.<br>• Important to consider whether measures reflect early life educational attainment or later life education and training.
**Income**	*Strengths*<br>• Captures materialist pathways to inequalities.
	Limitations<br>• Older people no longer in paid employment may be income-poor but asset rich.<br>• Income does not capture wealth accumulated over time through housing assets and other financial resources (e.g. savings).<br>• Potential difficulties collecting data where there are multiple income sources, and due to sensitivities of disclosing this type of information.<br>• Family and household measures of income assumes older people draw upon and benefit from the economic resources of younger family members, yet the reverse may also be true.<br>• Family and household measures assume older people share equal access to this resource: evidence indicates income sharing within households is not equal but varies according to numerous factors.<br>• Measures that include spousal income assume this resource is equally shared when this may not occur.
**Combined wealth/assets**	*Strengths*<br>• Captures a range of older people's sources of wealth and economic resources, including those accumulated over the life course.<br>• Measures accounting for outgoings (net) may provide a more accurate economic profile of older people.
	Limitations<br>• Data may be difficult to obtain for the same reasons as for *income*.
**Occupational class/ employment**	*Strengths*<br>• Easy to obtain and widely available in cohort datasets.
	Limitations<br>• Poor applicability to a largely retired population.<br>• Although considered a proxy for lifetime earnings, longest held or main occupation is not necessarily a reflection of later life advantage due to compounding role of health/ disability.<br>• May overlook older women, many of whom were absent from labour workforce at working age, and/or have interrupted employment histories due to child-rearing and caring roles.<br>• Employment 'status' that distinguishes only between those employed and not employed will not capture variations in disadvantage in older populations.
**Home ownership**	*Strengths*<br>• Captures a key component of older people's economic circumstance.
	Limitations<br>• Potentially a homogenous measure due to high levels of home ownership amongst older people in countries where home ownership is the norm.<br>• A dichotomised measure of ownership masks enormous regional differentials in accumulated housing wealth.

	• Home ownership may not signal accumulated wealth in countries where this is not the norm.<br>• Similar to income, captures only one aspect of older people's economic resources.
**Subjective measures**	*Strengths*<br>• May overcome limitations of objective measures in older populations (accessing sensitive data about a wide range of economic resources).
	Limitations<br>• Subjective assessments of economic circumstance are influenced by macro-economic factors (recessions income inequality, modernisation), undermining comparability of this measure over time and between countries where these conditions change/differ.<br>• Not clear to what extent subjective assessments represent a valid measure of socioeconomic position in later life.
**Area deprivation measures**	*Strengths*<br>• Area deprivation may give some indication of property value, an important component of accumulated wealth in older populations.<br>• Easy to obtain and widely available in datasets.<br>• May have value where area-level deprivation and social environment is thought to underlie health inequalities.
	Limitations<br>• Prone to ecological fallacy: those living in poor areas may not be poor themselves.<br>• Many area deprivation measures typically draw upon indicators more relevant to working age populations, although the Indices of Multiple Deprivation includes a sub-domain for older populations.
**House value**	*Strengths*<br>• Captures accumulated wealth over time for older populations.
	Limitations<br>• House value data may be difficult to collect if participants unwilling or unable to disclose, although approximate market valuations can be obtained independently.
**Household material deprivation**	*Strengths*<br>• Captures materialist pathways to inequality.<br>• Important when household environment is thought to contribute to poor health.
	Limitations<br>• Housing conditions may reflect availability of financial resources, which is only one aspect of older people's economic capital.
**Health insurance status**	*Strengths*<br>• May be a useful proxy indicator of income in the absence of income data.
	Limitations<br>• May be less appropriate in countries where health insurance is not widely used.<br>• Dichotomised response categories risk minimising substantial socioeconomic variation in older populations.
**Car ownership**	*Strengths*<br>• Easy data to obtain.
	Limitations<br>• Car ownership signals more than material resources and is compounded by the health and independence of the individual.
**Geography profile of residence**	*Strengths*<br>• Easy data to obtain.
	Limitations<br>• On its own, geographical profile of residence (e.g. urban/rural) unlikely to capture variations in socioeconomic inequalities.<br>• May be more relevant in countries where clear socioeconomic inequalities existing between rural and urban areas.<br>• Unclear to what extent this represents a valid measure of socioeconomic position.
**Living arrangements (alone/with others)**	*Limitations*

	• Unclear what pathway to socioeconomic inequality is captured by this measure.<br>• Unclear how this measure would accommodate those living in residential care with/without nursing.
**Proportion of life working part time**	*Strengths*<br>• May give some proxy indication of accumulated financial resources.
	Limitations<br>• May overlook older women, many of whom were absent from labour workforce at working age, and/or have interrupted employment histories due to child-rearing and caring roles.
**Marital status**	*Limitations*<br>• Unclear what pathway to socioeconomic inequality is captured by this measure.<br>• For the oldest old, populations may be biased towards widowed status.
**Perceived access to healthcare**	*Limitations*<br>• Unclear what pathway to socioeconomic inequality is captured by this measure.
**Out of pocket payments for healthcare**	*Limitations*<br>• Only appropriate in context of non-universal care systems.<br>• Unclear whether out of pocket payments reflects advantage (greater ability to pay for care) or disadvantage (having poorer health insurance coverage).
**Poverty Income Ratio/ threshold status/income as % of federal poverty level**	*Strengths*<br>• May be easier to access than income data.<br>• Could be used as a proxy for unavailable income data.
	Limitations<br>• Dichotomised response measure may mask substantial socioeconomic differences among older populations.<br>• When based on income, faces the same challenges as direct measures of income (i.e. captures only one aspect of older people's financial capital)

Table S3. Split of study samples between categories of educational attainment and home ownership

Study	Split of sample by categories of education (%)	Split of sample by categories of home ownership (%)	Country
Adjei 2017	US<br>Male/Female<br>Incomplete Secondary school or less: 21.5/21.3<br>Secondary completed: 31.7/38.4<br>Tertiary Completed or above: 46.9/40.3<br><br>UK<br>Male/Female<br>Incomplete secondary school or less: 63.3/76.5<br>Secondary completed: 18.5/13.5<br>Tertiary completed or above: 18.3/10.0<br><br>Italy<br>Male/Female<br>Incomplete sec. or less: 67.5/80.1<br>Secondary completed: 27.7/17.9<br>Tertiary completed or above: 4.8/2.1<br><br>Spain<br>Male/Female<br>Incomplete sec. or less: 69.3/77.7<br>Secondary completed: 23.2/18.5<br>Tertiary completed or above: 8.5/3.9<br><br>Germany<br>Male/Female<br>Incomplete Sec. or less: 10.7/28.9<br>Secondary completed: 41.8/53.6<br>Tertiary Completed or above: 47.5/17.5	US<br>Home owner: 84.3<br>Non-home owner: 15.7<br><br>UK<br>Home owner: 72.6<br>Non-home owner: 27.4<br><br>Italy<br>Home owner: 83.1<br>Non-home owner: 16.9<br><br>Spain<br>Home owner: 90.1<br>Non-home owner: 9.9<br><br>Germany<br>Home owner: 58.9<br>Non-home owner: 41.1	Multiple
Ahn 2012	<High school: 18.6<br>High school: 34.3<br>>High school: 47.1	NA	US
Aida 2011	<6 years: 3.5<br>6-9 years: 50.5<br>10-12 years: 33.7<br>13+ years: 12.3	NA	Japan
Allen 2011	Rural/urban<br>< Secondary school: 31.1/32.9<br>Secondary school graduation: 10.9/17.0<br>Some post-secondary school education: 10.1/7.3<br>Post secondary degree/diploma: 47.9/42.9	NA	Canada
Ament 2012	High education level: 10.9<br>Low education level: 89.1	NA	Netherlands
Auchincloss 2001	No high school: 22.5<br>Some high school: 15.9<br>High school degree: 35.8<br>College: 25.8	NA	US

Cain 2017	No high school diploma: 17.0<br>HS graduate: 25.0<br>Some college—no degree: 25.0<br>College degree (undergraduate): 18.0<br>Grad or prof degree: 15.0	NA	US
Connelly 2010	Data not extracted due to volume. Reader is referred to original publication.	Owner: 71.0<br>Renter: 29.0	Northern Ireland
Dalstra 2006	Not reported due to volume. Reader is referred to table 2 of original publication. Authors note that across countries, the proportion of participants was typically larger in the lowest educational attainment categories.	Not reported, but authors note that there was much variability between countries in the distribution of participants between home owners and renters.	Europe
Enroth 2013	Male/Female:<br>High educated: 20.0/11.0<br>Middle educated: 30.0/17.0<br>Low educated: 47.0/68.0<br>Education unknown: 3.0/4.0	NA	US
Enroth 2019	Sweden<br>Basic: 47.2<br>Higher: 52.8<br><br>Norway<br>Basic: 33.2<br>Higher: 66.8<br><br>Denmark<br>Basic: 47.7<br>Higher: 52.3	NA	Sweden, Denmark, Norway
Fernandez-Martinez 2000	< Elementary school: 31.6<br>Elementary school: 38.1<br>Middle/high school or higher: 30.3	NA	Spain
Fernandez-Mayorales 2000	Higher studies: 5.0<br>Secondary: 49.5<br>< Primary: 45.5	NA	Spain
Fors 2015	1992<br>Grade school or less: 76.9<br>Beyond grade school: 23.1<br><br>2002<br>Grade school or less: 68.2<br>Beyond grade school: 38.1<br><br>2011<br>Grade school or less: 57.7<br>Beyond grade school:42.3	NA	Sweden
Francois 2011	No info: 3.9<br>No degree or primary: 34.7<br>Lower secondary: 24.4<br>Higher secondary: 21.7<br>Higher education: 15.3	NA	Belgium

Franse 2017	Tertiary: 10.5<br>Secondary: 56.7<br>Primary: 32.8	NA	Netherlands
Freedman 2004	Plans A & B/HMO enrolees/FFS enrolees<br>High school degree: 38.2/32.0/35.0<br>No degree: 61.8/68.0/65.0	NA	US
Giron 2012	Illiterate or no education: 37.1<br>Primary and secondary 1st cycle: 49.7<br>Second cycle secondary and post-secondary: 7.2<br>university: 6.1	NA	Spain
Gomez-Baya 2020	No studies 7.7<br>Primary 27.9<br>Secondary, professional training 30.8<br>University degree 30.4<br>University Post-degree 2.9<br>No answer 0.3	NA	Spain
Grau 2001	<High school: 24.0<br>High school: 41.0<br>Post-high school: 34.0	NA	US
Grundy 2007	NA	Home owner: 68.4<br>Social tenant: 25.6<br>Private tenant: 6.0	UK
Hancock 2002	NA	Home owner: 49.3<br>Non-home owner: 50.7	UK
Hoeck 2013	No information: 2.9<br>No degree or primary: 28.7<br>Lower secondary: 24.6<br>Higher secondary: 26.2<br>Higher education: 17.6	Home owner: 75.4<br>Non-home owner: 24.6	Belgium
Honjo 2006	13+ years: 34.0<br>12 years: 35.2<br>11 years: 30.8	NA	Japan
Howard 2006	High school degree: 64.2<br>No high school degree: 35.8	NA	US
Huijts 2010	Denmark, Men:<br>Primary: 16.0<br>Secondary: 44.9<br>Tertiary: 39.1<br><br>Denmark, Women:<br>Primary: 20.0<br>Secondary: 34.1<br>Tertiary: 45.1<br><br>Finland, Men:<br>Primary: 33.9<br>Secondary: 37.9<br>Tertiary: 28.3<br><br>Finland, Women:<br>Primary: 33.9	NA	Finland, Denmark

	Secondary: 29.4<br>Tertiary: 36/7		
Illoabuchi 2014	< 12 years of education: 67<br>> 12 years of education: 33	NA	US
Jenkins 2020	NA	Non-home owners: 11.7<br>Owner: 88.3	US
Kim 2011	Elementary school: 62.1<br>Middle school: 14.4<br>High school: 23.4	NA	Korea
Kim 2011	None: 8.57<br>1 - 11 years: 27.49<br>>12 years: 63.94	NA	Korea
Knurowski 2005	Basic or lower: 32.4<br>secondary: 45.6<br>University: 22.0	Home owners: 66.0<br>Non-home owner: 34.0	Poland
Lee 2020	< High school: 12.4<br>High school: 30.3<br>Attended college: 28.3<br>Graduated college: 29.0	NA	US
Lindenaur 2003	NA	Non-home owner: 37.7<br>Home owner: 62.3	US
Lopez-de-Andres 2018	EHSS 2009<br>No studies/primary: 84.7<br>Secondary: 10.3<br>Higher education: 5.0<br><br>EHSS 2014<br>No studies/primary: 82.5<br>Secondary: 10.0<br>Higher education: 7.5	NA	Spain
Luchetti 2010	Under 5 years: 24.5<br>Over 5 years: 75.5	NA	Italy
Lum 2004	No high school diploma: 43.4<br>High school diploma: 29.5<br>Some college: 14.8<br>College: 12.2	NA	US
Lupi-Pegurier 2011	< Baccalaureate: 33.8<br>Baccalaureate: 52.7<br>> Baccalaureate: 13.5	NA	France
Maniecka-Bryła 2011	Tertiary: 21.2<br>Secondary: 30.5<br>Vocational: 7.3<br>Primary: 41.0	NA	Poland
Martikainen 2008	Men<br>Basic: 79.1<br>Intermediate: 11.0<br>Tertiary: 9.9<br><br>Women<br>Basic: 78.4	Men<br>Owner: 67.8<br>Non-owner: 32.2<br><br>Women<br>Owner: 69.8<br>Non-owner: 30.2	Finland

	Intermediate: 13.3<br>Tertiary: 8.3		
Mather 2014	65-79<br>No school certificate: 14.8<br>School cert: 25.5<br>Higher school cert: 22.9<br>Cert or diploma: 18.9<br>University+: 18.0<br><br>80+<br>No school certificate: 18.3<br>School cert: 25.8<br>Higher school cert: 23.9<br>Cert or diploma: 16.9<br>University+: 15.1	NA	Australia
McCann 2011	NA	Renters: 28.0<br>Non-renters: 72.0	UK
Nicklett 2011	< High school: 76.5<br>High school: 14.3<br>Some college or more: 9.2	NA	US
Niefield 2005	0-8 years/don't know: 51.0<br>9-13+ years: 49.0	Home owner: 20.0<br>Non-home owner: 80.0	US
Nieman 2014	< High school: 11.8<br>Some high school 15.7<br>High school graduate 29.4<br>Some college or associates degree 23.7<br>College graduate or above 19.4	NA	US
Nihtila 2007	Female/Male<br>Tertiary: 8.0/13.6<br>Intermediate: 13.6/12.3<br>Basic or less: 78.4/74.1	Female/Male<br>Owner: 78.1/83.8<br>Renter: 18.0/12.7<br>Other or unknown: 3.8/3.5	Finland
Orfila 2000	65-74<br>High school or university: 26.5<br>Primary school: 61.9<br>Unable to read or write: 11.7<br><br>72-79<br>High school or university: 20.9<br>Primary school: 72.9<br>Unable to read or write: 6.2<br><br>80+<br>High school or university: 13.6<br>Primary school: 79.7<br>Unable to read or write: 6.8	NA	Spain
Low 2009	< Secondary School: 40.4%<br>Secondary graduates: 13.1%<br>Post secondary education: 43.5%	NA	Canada
Park 2014	Male/Female<br>Primary school: 47.4/84.9<br>Middle school: 17.0/8.7	NA	Korea

	High school: 22.0/5.1<br>College+: 14.0/1.3		
Prajsner 2015 & 2016	No education: 1.4<br>Primary incomplete: 9.5<br>Primary: 39.1<br>Vocational: 17.5<br>Secondary: 21.6<br>Higher: 10.9	NA	Poland
Reyes-Oritz 2010	0-5 years: 51.2<br>5+ years: 48.8	NA	US
Roe-Prior 2007	<High school: 42.0<br>High school diploma: 31.0<br>Post high school: 29.0	NA	US
Rostad 2009	>12 years: 4.0<br>8-11 years: 27.0<br><7 years: 69.0	NA	Norway
Rueda 2008	Male/Female<br>Without formal education: 6.5/9.9<br>Primary education or less: 31.5/34.5<br>Secondary education: 43.2/44.5<br>Higher than secondary education: 17.9/9.8	NA	Spain
Rueda 2009	Women:<br>> Primary schooling: 17.8<br>Primary: 30.7<br>< Primary: 51.5<br><br>Men:<br>> Primary schooling: 30.2<br>Primary: 33.8<br>< Primary: 36.0	NA	Spain
Rueda 2012	BASQUE<br>Primary +: 40.2/22.4<br>Primary: 49.0/61.5<br><Primary: 10.8/16.1<br><br>NAVARRA<br>Primary +: 17.5/10.9<br>Primary: 59.5/64.5<br><Primary: 23.0/24.6<br><br>ANDALUSIA<br>Primary +: 16.9/8.4<br>Primary: 35.5/29.4<br><Primary: 47.6/62.2<br><br>MURCIA<br>Primary +: 17.3/4.5<br>Primary: 34.6/29.9<br><Primary: 48.1/65.6	NA	Spain
Shea 2003	US<br>< High school: 35.4	NA	US

	Some high school: 42.2<br>Some college: 22.4<br><br>Sweden<br>< High school: 68.2<br>Some high school: 14.3<br>Some college: 17.5		
Sherman 2012	Elementary: 49.0<br>Upper sec: 29.0<br>University: 20.0<br>Missing: 2.0	NA	Sweden
Sulander 2012	Male/Female<br>Secondary: 58.8/46.0<br>Middle: 18.8/29.8<br>Elementary: 22.4/24.3	NA	Finland
Suominen-Taipale 2004	Primary school: 49.0<br>Middle level: 25.0<br>University: 8.0	NA	Norway
Suominen-Taipale 2004	Primary school: 57.0<br>Middle level: 30.0<br>University: 8.0	NA	Finland
Tigani 2012	Illiterate: 42.8<br>Unfinished primary: 30.8<br>Primary: 14.0<br>Unfinished secondary: 3.3<br>Secondary: 4.5<br>Unfinished tertiary: 0.5<br>Tertiary: 3.5	NA	Greece
Tomiak 2000	Male/Female, Years in Education<br>Quartile 1: 24.1/22.3<br>Quartile 2: 26.2/27.4<br>Quartile 3: 23.9/24.2<br>Quartile 4: 25.8/26.1	Male/Female<br>Home owner: 77.9/64.1<br>Non-home owner: 22.1/35.9	Canada
Trachte 2016	Men:<br>High: 27.6<br>Medium: 12.5<br>Low: 59.9<br><br>Women:<br>High: 11.5<br>Medium: 18.6<br>Low: 69.9	NA	Germany
	0-9 years: 9.4<br>10-12 years: 44.9<br>13+ years: 45.7	Home owner: 82.5<br>Non-home owner: 17.7	US
von dem Knesebeck 2003	0-9 years: 54.3<br>10-12 years: 27.5<br>13+ years: 18.2	Home owner: 54.0<br>Non-home owner: 46.00	Germany
von dem Knesebeck 2015	Inadequately completed general education: 1.3<br>General elementary education: 13.6<br>Basic vocational qualification or general elementary	NA	Germany

	education and vocational qualification: 46.0<br>Intermediate general qualification: 2.5<br>Intermediate vocational or intermediate general qualification and vocational qualification: 19.9<br>General maturity certificate: 1.1<br>Vocational maturity certificate/general maturity certificate and vocational qualification: 4.0<br>Lower tertiary education: 4.8<br>Higher tertiary education: 7.0		
Wastesson 2014	Low: 56.5<br>Medium: 28.1<br>High: 15.4	NA	Sweden

BMJ Publishing Group Limited (BMJ) disclaims all liability and responsibility arising from any reliance placed on this supplemental material which has been supplied by the author(s)
