# Supplementary material for: Measuring older people’s socioeconomic position: a scoping review of studies of self-rated health, health service and social care use
Source: J Epidemiol Community Health. 2022 Mar 15;76(6):572–9. doi: 10.1136/jech-2021-218265 (PMC9118079; doi:10.1136/jech-2021-218265)
Supplement: Supplementary data [file jech-2021-218265supp002.pdf]

References of studies included in the review[1-138]

1. Aschan-Leygonie C, Baudet-Michel S, Mathian H, Sanders L. Gaining a better understanding of respiratory health inequalities among cities: an ecological case study on elderly males in the larger French cities. *Int J Health Geogr* 2013; **12**: 19.
2. Assari S, Cobb S, Saqib M, Bazargan M. Economic Strain Deteriorates While Education Fails to Protect Black Older Adults Against Depressive Symptoms, Pain, Self-rated Health, Chronic Disease, and Sick Days. *J Ment Health Clin Psychol* 2020; **4**(2): 49-62.
3. Cain CL, Wallace SP, Ponce NA. Helpfulness, Trust, and Safety of Neighborhoods: Social Capital, Household Income, and Self-Reported Health of Older Adults. *Gerontologist*; **58**(1): 4-14.
4. Connolly S, O'Reilly D, Rosato M. House value as an indicator of cumulative wealth is strongly related to morbidity and mortality risk in older people: a census-based cross-sectional and longitudinal study. *International Journal of Epidemiology* 2010; **39**(2): 383-91.
5. Dalstra JA, Kunst AE, Mackenbach JP, Health EUWGoSIi. A comparative appraisal of the relationship of education, income and housing tenure with less than good health among the elderly in Europe. *Soc Sci Med* 2006; **62**(8): 2046-60.
6. Elovainio M, Kivimaki M. Sense of coherence and social support - Resources for subjective well-being and health of the aged in Finland. *International Journal of Social Welfare* 2000; **9**(2): 128-35.
7. Fors S, Thorslund M. Enduring inequality: educational disparities in health among the oldest old in Sweden 1992-2011. *Int J Public Health*; **60**(1): 91-8.
8. Franse CB, van Grieken A, Qin L, Melis RJF, Rietjens JAC, Raat H. Socioeconomic inequalities in frailty and frailty components among community-dwelling older citizens. *PLoS One* 2017; **12**(11): e0187946.
9. Fukuda Y, Nakamura K, Takano T, Nakao H, Imai H. Socioeconomic status and cancer screening in Japanese males: Large inequlaity in middle-aged and urban residents. *Environ Health Prev Med* 2007; **12**(2): 90-6.
10. Gomez-Baya D, Salinas-Perez JA, Rodero-Cosano ML, Alvarez-Galvez J. Socioeconomic Inequalities in Health Through Lifestyles: Analysing Gender and Age Differences in Andalusia, Spain. *J Community Health* 2020; **45**(4): 836-45.
11. Grundy E, Sloggett A. Health inequalities in the older population: the role of personal capital, social resources and socio-economic circumstances. *Soc Sci Med* 2003; **56**(5): 935-47.
12. Hamada S, Takahashi H, Sakata N, et al. Household Income Relationship With Health Services Utilization and Healthcare Expenditures in People Aged 75 Years or Older in Japan: A Population-Based Study Using Medical and Long-term Care Insurance Claims Data. *J Epidemiol* 2019; **29**(10): 377-83.
13. Hardy D, Chan W, Liu CC, et al. Racial disparities in the use of hospice services according to geographic residence and socioeconomic status in an elderly cohort with nonsmall cell lung cancer. *Cancer* 2011; **117**(7): 1506-15.
14. Hoebel J, Rommel A, Schroder SL, Fuchs J, Nowossadeck E, Lampert T. Socioeconomic Inequalities in Health and Perceived Unmet Needs for Healthcare among the Elderly in Germany. *Int J Environ Res Public Health* 2017; **14**(10).
15. Honjo K, Kawakami N, Takeshima T, et al. Social class inequalities in self-rated health and their gender and age group differences in Japan. *J Epidemiol* 2006; **16**(6): 223-32.
16. Howard DH, Sentell T, Gazmararian JA. Impact of health literacy on socioeconomic and racial differences in health in an elderly population. *J Gen Intern Med* 2006; **21**(8): 857-61.

17. Huang Y, Meyer P, Jin L. Neighborhood socioeconomic characteristics, healthcare spatial access, and emergency department visits for ambulatory care sensitive conditions for elderly. *Prev Med Rep* 2018; **12**: 101-5.
18. Ichida Y, Kondo K, Hirai H, Hanibuchi T, Yoshikawa G, Murata C. Social capital, income inequality and self-rated health in Chita peninsula, Japan: a multilevel analysis of older people in 25 communities. *Soc Sci Med* 2009; **69**(4): 489-99.
19. Ilinca S, Rodrigues R, Schmidt AE. Fairness and Eligibility to Long-Term Care: An Analysis of the Factors Driving Inequality and Inequity in the Use of Home Care for Older Europeans. *Int J Environ Res Public Health* 2017; **14**(10).
20. Iloabuchi TC, Mi D, Tu W, Counsell SR. Risk factors for early hospital readmission in low-income elderly adults. *J Am Geriatr Soc* 2014; **62**(3): 489-94.
21. Jenkins CL. Resource effects on access to long-term care for frail older people. *J Aging Soc Policy* 2001; **13**(4): 35-52.
22. Jenkins Morales M, Robert SA. The Effects of Housing Cost Burden and Housing Tenure on Moves to a Nursing Home Among Low- and Moderate-Income Older Adults. *Gerontologist* 2020; **60**(8): 1485-94.
23. Jiang P, Babazono A, Fujita T. Health Inequalities Among Elderly Type 2 Diabetes Mellitus Patients in Japan. *Popul Health Manag* 2020; **23**(3): 264-70.
24. Jyvakorpi SK, Urtamo A, Strandberg TE. Self-Perception of Economic Means Is Associated with Dietary Choices, Diet Quality and Physical Health in the Oldest Old Men from the Highest Socioeconomic Group. *J Nutr Health Aging* 2019; **23**(1): 60-2.
25. Kim CB, Yoon SJ, Ko J. Economic Activity and Health Conditions in Adults Aged 65 Years and Older: Findings of the Korean National Longitudinal Study on Aging. *Healthcare (Basel)* 2017; **5**(4).
26. Kim H, Lyons AC. No pain, no strain: Impact of health on the financial security of older Americans. *Journal of Consumer Affairs* 2008; **42**(1): 9-36.
27. Kiuchi S, Aida J, Kusama T, et al. Does public transportation reduce inequalities in access to dental care among older adults? Japan Gerontological Evaluation Study. *Community Dent Oral Epidemiol*; **48**(2): 109-18.
28. Lopez-de-Andres A, de Miguel-Diez J, Hernandez-Barrera V, et al. Effect of the economic crisis on the use of health and home care services among elderly Spanish diabetes patients. *Diabetes Res Clin Pract* 2018; **140**: 27-35.
29. Lum T. Health-wealth association among older Americans: Racial and ethnic differences. *Social Work Research* 2004; **28**(2): 105-16.
30. Lupi-Pegurier L, Clerc-Urmes I, Abu-Zaineh M, Paraponaris A, Ventelou B. Density of dental practitioners and access to dental care for the elderly: a multilevel analysis with a view on socio-economic inequality. *Health Policy* 2011; **103**(2-3): 160-7.
31. Martikainen P, Nihtila E, Moustgaard H. The effects of socioeconomic status and health on transitions in living arrangements and mortality: a longitudinal analysis of elderly Finnish men and women from 1997 to 2002. *J Gerontol B Psychol Sci Soc Sci* 2008; **63**(2): S99-109.
32. McFadden E, Luben R, Bingham S, Wareham N, Kinmonth AL, Khaw KT. Social inequalities in self-rated health by age: cross-sectional study of 22,457 middle-aged men and women. *BMC Public Health* 2008; **8**: 230.
33. McMunn A, Nazroo J, Breeze E. Inequalities in health at older ages: a longitudinal investigation of the onset of illness and survival effects in England. *Age Ageing* 2009; **38**(2): 181-7.
34. Mishra GD, Ball K, Dobson AJ, Byles JE. Do socioeconomic gradients in women's health widen over time and with age? *Social science & medicine (1982)* 2004; **58**(9): 1585-95.
35. Muckenhuber J, Fern, ez K, et al. Trends in inequalities in health, risk and preventive behaviour among the advanced-age population in Austria: 1983-2007. *PLoS One* 2014; **9**(5): e97400.
36. Munford LA, Sidaway M, Blakemore A, Sutton M, Bower P. Associations of participation in community assets with health-related quality of life and healthcare usage: a cross-sectional study of older people in the community. *BMJ Open* 2017; **7**(2): e012374.

BMJ Publishing Group Limited (BMJ) disclaims all liability and responsibility arising from any reliance placed on this supplemental material which has been supplied by the author(s)

37. Murata F, Babazono A, Fukuda H. Effect of income on length of stay in a hospital or long-term care facility among older adults with dementia in Japan. *Int J Geriatr Psychiatry* 2020; **35**(3): 302-11.

38. Nicklett EJ. Socioeconomic status and race/ethnicity independently predict health decline among older diabetics. *BMC Public Health* 2011; **11**: 684.

39. Niefeld MR, Kasper JD. Access to ambulatory medical and long-term care services among elderly Medicare and Medicaid beneficiaries: organizational, financial, and geographic barriers. *Med Care Res Rev* 2005; **62**(3): 300-19.

40. Nieman CL, Marrone N, Szanton SL, Thorpe R, J., Lin FR. Racial/Ethnic and Socioeconomic Disparities in Hearing Care among Older Americans. *Otolaryngology–Head and Neck Surgery* 2014; **151**(1): P225-P.

41. Nihtila E, Martikainen P. Why older people living with a spouse are less likely to be institutionalized: the role of socioeconomic factors and health characteristics. *Scand J Public Health* 2008; **36**(1): 35-43.

42. Park S, Kim B, Kwon E, Kown G. Influence of senior housing types on cognitive decline and nursing home admission among lower-income older adults. *Aging Ment Health* 2020; **24**(10): 1579-88.

43. Patel R, Lawlor DA, Ebrahim S. Socio-economic position and the use of preventive health care in older British women: A cross-sectional study using data from the British Women's Heart and Health Study cohort. *Family Practice* 2007; **24**(1): 7-10.

44. Pirani E, Salvini S. Place of living and health inequality: a study for elderly Italians. *Statistical Methods and Applications* 2012; **21**(2): 211-26.

45. Prajsner A, Chudek J, Szybalska A, et al. Socioeconomic profile of elderly Polish men treated for benign prostate hyperplasia: Results of the population-based PolSenior study. *European Geriatric Medicine* 2015; **6**(1): 53-7.

46. Prajsner A, Chudek J, Szybalska A, et al. Socioeconomic determinants of prostate-specific antigen testing and estimation of the prevalence of undiagnosed prostate cancer in an elderly Polish population based on the PolSenior study. *Arch Med Sci* 2016; **12**(5): 1028-35.

47. Ramsay SE, Papachristou E, Watt RG, et al. Socioeconomic disadvantage across the life-course and oral health in older age: findings from a longitudinal study of older British men. *J Public Health (Oxf)* 2018; **40**(4): e423-e30.

48. Reyes-Ortiz CA, Markides KS. Socioeconomic factors, immigration status, and cancer screening among Mexican American women aged 75 and older. *Health Care Women Int* 2010; **31**(12): 1068-81.

49. Robert SA, Li LW. Age variation in the relationship between community socioeconomic status and adult health. *Research on Aging* 2001; **23**(2): 233-58.

50. Rostad B, Deeg DJH, Schei B. Socioeconomic inequalities in health in older women. *Eur J Ageing* 2009; **6**(1): 39-47.

51. Rueda S, Artazcoz L. Gender inequality in health among elderly people in a combined framework of socioeconomic position, family characteristics and social support. *Ageing & Society* 2009; **29**(4): 625-47.

52. Schmitz AL, Pfortner TK. Health inequalities in old age: the relative contribution of material, behavioral and psychosocial factors in a German sample. *J Public Health (Oxf)* 2018; **40**(3): e235-e43.

53. Shebehe J, Hansson A. High hospital readmission rates for patients aged >/=65 years associated with low socioeconomic status in a Swedish region: a cross-sectional study in primary care. *Scand J Prim Health Care*; **36**(3): 300-7.

54. Sigurdardottir AK, Kristofersson GK, Gustafsdottir SS, et al. Self-rated health and socio-economic status among older adults in Northern Iceland. *International Journal of Circumpolar Health* 2019; **78**(1): 1697476.

55. Stone J, Evandrou M, Falkingham J, Vlachantoni A. Women's economic activity trajectories over the life course: implications for the self-rated health of women aged 64+ in England. *J Epidemiol Community Health* 2015; **69**(9): 873-9.
56. Sulander T, Rahkonen O, Nummela O, Uutela A. Ten year trends in health inequalities among older people, 1993-2003. *Age Ageing* 2009; **38**(5): 613-7.
57. Torssander J, Ahlbom A, Modig K. Four Decades of Educational Inequalities in Hospitalization and Mortality among Older Swedes. *PLoS One* 2016; **11**(3): e0152369.
58. Trachte F, Geyer S, Sperlich S. Impact of physical activity on self-rated health in older people: do the effects vary by socioeconomic status? *J Public Health (Oxf)* 2016; **38**(4): 754-9.
59. Van Ourti T. Socio-economic inequality in ill-health amongst the elderly. Should one use current or permanent income? *Journal of health economics* 2003; **22**(2): 219-41.
60. von dem Knesebeck O, Bickel H, Fuchs A, et al. Social inequalities in patient-reported outcomes among older multimorbid patients--results of the MultiCare cohort study. *Int J Equity Health* 2015; **14**(1): 17.
61. Wang S, Hoshi T, Ai B. Does Social Interaction Explain Socioeconomic Inequalities in Health Status? Results from the Japanese Community-Dwelling Elderly Age 65 to 84 Years. *Educational Gerontology* 2014; **41**(2): 81-92.
62. Wastesson JW, Fors S, Parker MG, Johnell K. Inequalities in health care use among older adults in Sweden 1992-2011: a repeated cross-sectional study of Swedes aged 77 years and older. *Scand J Public Health* 2014; **42**(8): 795-803.
63. Williams BA, Lindquist K, Sudore RL, Covinsky KE, Walter LC. Screening mammography in older women. Effect of wealth and prognosis. *Arch Intern Med* 2008; **168**(5): 514-20.
64. Adjei NK, Brand T, Zeeb H. Gender inequality in self-reported health among the elderly in contemporary welfare countries: A cross-country analysis of time use activities, socioeconomic positions and family characteristics. *PLoS One* 2017; **12**(9): e0184676.
65. Ahn S, Hochhalter AK, Moudouni DK, Smith ML, Ory MG. Self-reported physical and mental health of older adults: the roles of caregiving and resources. *Maturitas* 2012; **71**(1): 62-9.
66. Aida J, Kondo K, Kondo N, Watt RG, Sheiham A, Tsakos G. Income inequality, social capital and self-rated health and dental status in older Japanese. *Soc Sci Med* 2011; **73**(10): 1561-8.
67. Allan DE, Funk LM, Reid RC, Cloutier-Fisher D. Exploring the influence of income and geography on access to services for older adults in British Columbia: a multivariate analysis using the Canadian Community Health Survey (Cycle 3.1). *Can J Aging* 2011; **30**(1): 69-82.
68. Alwan N, Wilkinson M, Birks D, Wright J. Do Standard Measures of Deprivation Reflect Health Inequalities in Older People? *Journal of Public Health Policy* 2007; **28**(3): 356-62.
69. Angel RJ, Frisco M, Angel JL, Chiriboga DA. Financial strain and health among elderly Mexican-origin individuals. *Journal of Health and Social Behavior* 2003; **44**(4): 536-51.
70. Antonelli-Incalzi R, Ancona C, Forastiere F, Belleudi V, Corsonello A, Perucci CA. Socioeconomic status and hospitalization in the very old: a retrospective study. *BMC Public Health* 2007; **7**: 227.
71. Bambra C, Netuveli G, Eikemo TA. Welfare state regime life courses: The development of Western European welfare state regimes and age-related patterns of educational inequalities in self-reported health. *The financial and economic crises and their impact on health and social well-being* 2014: 316-36.
72. Broese van Groenou M, Glaser K, Tomassini C, Jacobs T. Socio-economic status differences in older people's use of informal and formal help: a comparison of four European countries. *Ageing and Society* 2006; **26**(5): 745-66.
73. Cohen D, Manuel DG, Tugwell P, Ramsay T, Sanmartin C. Inequity in primary and secondary preventive care for acute myocardial infarction? Use by socioeconomic status across middle-aged and older patients. *Can J Cardiol* 2013; **29**(12): 1579-85.

74. Evans GW, Wethington E, Coleman M, Worms M, Frongillo EA. Income health inequalities among older persons: the mediating role of multiple risk exposures. *J Aging Health* 2008; **20**(1): 107-25.
75. Fernandez-Martinez B, Prieto-Flores ME, Forjaz MJ, Fernandez-Mayoralas G, Rojo-Perez F, Martinez-Martin P. Self-perceived health status in older adults: regional and sociodemographic inequalities in Spain. *Revista de saude publica* 2012; **46**(2): 310-9.
76. Gill G, Blackmore K, P Geraghty D, FitzGerald D. The impact of residential socio-economic profile on medical service utilisation and nursing home attendance claims by older Australians. *Australian Journal of Primary Health* 2004; **10**(3): 137-43.
77. Grau L, West B, Gregory P, Marcella S. The effects of socioeconomic and socioenvironmental factors on the self-reported health of elderly blacks and whites. *N J Med* 2001; **98**(8): 33-9.
78. Hancock R, Arthur A, Jagger C, Matthews R. The effect of older people's economic resources on care home entry under the United Kingdom's long-term care financing system. *J Gerontol B Psychol Sci Soc Sci* 2002; **57**(5): S285-93.
79. Hoeck S, Francois G, Van der Heyden J, Geerts J, Van Hal G. Healthcare utilisation among the Belgian elderly in relation to their socio-economic status. *Health Policy* 2011; **99**(2): 174-82.
80. Hoeck S, van der Heyden J, Geerts J, Van Hal G. Preventive care use among the Belgian elderly population: does socio-economic status matter? *Int J Environ Res Public Health*; **11**(1): 355-72.
81. Kim D, Shin H, Kim C-y. Equitable Access to Health Care for the Elderly in South Korea. *Research on Aging* 2011; **34**(4): 475-96.
82. Kim J. The mediating effects of lifestyle factors on the relationship between socioeconomic status and self-rated health among middle-aged and older adults in Korea. *Int J Aging Hum Dev* 2011; **73**(2): 153-73.
83. von dem Knesebeck O, Lüschen G, Cockerham WC, Siegrist J. Socioeconomic status and health among the aged in the United States and Germany: A comparative cross-sectional study. *Social Science & Medicine* 2003; **57**(9): 1643-52.
84. Knurowski T, van Dijk JP, Geckova AM, Brzyski P, Tobiasz-Adamczyk B, van den Heuvel WJ. Socio-economic health differences among the elderly population in Krakow, Poland. *Soz Praventivmed* 2005; **50**(3): 177-85.
85. Li Y, Robert SA. The Contributions of Race, Individual Socioeconomic Status, and Neighborhood Socioeconomic Context on the Self-Rated Health Trajectories and Mortality of Older Adults. *Research on Aging* 2008; **30**(2): 251-73.
86. Mather T, Banks E, Joshy G, Bauman A, Phongsavan P, Korda RJ. Variation in health inequalities according to measures of socioeconomic status and age. *Aust N Z J Public Health* 2014; **38**(5): 436-40.
87. Nihtila E, Martikainen P. Household income and other socio-economic determinants of long-term institutional care among older adults in Finland. *Popul Stud (Camb)* 2007; **61**(3): 299-314.
88. Otaki N, Tanino N, Yokoro M, et al. Relationship between Economic Security and Self-Rated Health in Elderly Japanese Residents Living Alone. *J Nutr Health Aging* 2018; **22**(6): 695-9.
89. Park BH, Jung M, Lee TJ. Associations of income and wealth with health status in the Korean elderly. *J Prev Med Public Health* 2009; **42**(5): 275-82.
90. Pirani E, Salvini S. Socioeconomic Inequalities and Self-Rated Health: A Multilevel Study of Italian Elderly. *Population Research and Policy Review* 2012; **31**(1): 97-117.
91. Piumatti G. Relations between longitudinal trajectories of subjective financial wellbeing with self-rated health among elderly. *Medicina (Kaunas)* 2017; **53**(5): 323-30.
92. Rathore SS, Masoudi FA, Wang Y, et al. Socioeconomic status, treatment, and outcomes among elderly patients hospitalized with heart failure: findings from the National Heart Failure Project. *Am Heart J* 2006; **152**(2): 371-8.

93. Robert SA, Cherepanov D, Palta M, Dunham NC, Feeny D, Fryback DG. Socioeconomic status and age variations in health-related quality of life: results from the national health measurement study. *J Gerontol B Psychol Sci Soc Sci* 2009; **64**(3): 378-89.
94. Robert SA, Lee KY. Explaining race differences in health among older adults - The contribution of community socioeconomic context. *Research on Aging* 2002; **24**(6): 654-83.
95. Rodrigues R, Ilinca S, Schmidt AE. Income-rich and wealth-poor? The impact of measures of socio-economic status in the analysis of the distribution of long-term care use among older people. *Health Econ*; **27**(3): 637-46.
96. Rueda S. Health inequalities among older adults in Spain: the importance of gender, the socioeconomic development of the region of residence, and social support. *Womens Health Issues* 2012; **22**(5): e483-90.
97. Rueda S, Artazcoz L, Navarro V. Health inequalities among the elderly in western Europe. *J Epidemiol Community Health* 2008; **62**(6): 492-8.
98. Schmidt AE. Analysing the importance of older people's resources for the use of home care in a cash-for-care scheme: evidence from Vienna. *Health & Social Care in the Community* 2017; **25**(2): 514-26.
99. Sheifer SE, Rathore SS, Gersh BJ, et al. Time to presentation with acute myocardial infarction in the elderly: associations with race, sex, and socioeconomic characteristics. *Circulation* 2000; **102**(14): 1651-6.
100. Sulander T, Pohjolainen P, Karvinen E. Self-rated health (SRH) and socioeconomic position (SEP) among urban home-dwelling older adults. *Arch Gerontol Geriat* 2012; **54**(1): 117-20.
101. Wachelder JJH, van Drunen I, Stassen PM, et al. Association of socioeconomic status with outcomes in older adult community-dwelling patients after visiting the emergency department: a retrospective cohort study. *BMJ Open* 2017; **7**(12): e019318.
102. Walker A, Pearse J, Thurecht L, Harding A. Hospital admissions by socio-economic status: does the 'inverse care law' apply to older Australians? *Aust N Z J Public Health*; **30**(5): 467-73.
103. Allin S, Masseria C, Mossialos E. Measuring socioeconomic differences in use of health care services by wealth versus by income. *Am J Public Health* 2009; **99**(10): 1849-55.
104. Auchincloss AH, Van Nostrand JF, Ronsaville D. Access to health care for older persons in the United States: personal, structural, and neighborhood characteristics. *J Aging Health* 2001; **13**(3): 329-54.
105. Enroth L, Raitanen J, Hervonen A, Jylha M. Do socioeconomic health differences persist in nonagenarians? *J Gerontol B Psychol Sci Soc Sci* 2013; **68**(5): 837-47.
106. Fernandez-Mayoralas G, Rodriguez V, Rojo F. Health services accessibility among Spanish elderly. *Social Science & Medicine* 2000; **50**(1): 17-26.
107. Freedman VA, Rogowski J, Wickstrom SL, Adams J, Marainen J, Escarce JJ. Socioeconomic disparities in the use of home health services in a medicare managed care population. *Health Serv Res* 2004; **39**(5): 1277-97.
108. Giron P. Is age associated with self-rated health among older people in Spain? *Central European journal of public health* 2012; **20**(3): 185-90.
109. Lasheras C, Patterson AM, Casado C, Fernandez S. Effects of education on the quality of life, diet, and cardiovascular risk factors in an elderly Spanish community population. *Experimental aging research* 2001; **27**(3): 257-70.
110. Nummela OP, Sulander TT, Heinonen HS, Uutela AK. Self-rated health and indicators of SES among the ageing in three types of communities. *Scandinavian Journal of Public Health* 2007; **35**(1): 39-47.
111. Orfila F, Ferrer M, Lamarca R, Alonso J. Evolution of self-rated health status in the elderly: cross-sectional vs. longitudinal estimates. *J Clin Epidemiol* 2000; **53**(6): 563-70.
112. Park JM. Health status and health services utilization in elderly Koreans. *Int J Equity Health* 2014; **13**: 73.

113. Sherman H, Forsberg C, Karp A, Tornkvist L. The 75-year-old persons' self-reported health conditions: a knowledge base in the field of preventive home visits. *J Clin Nurs* 2012; **21**(21-22): 3170-82.
114. Suominen-Taipale AL, Koskinen S, Martelin T, Holmen J, Johnsen R. Differences in older adults' use of primary and specialist care services in two Nordic countries. *European Journal of Public Health* 2004; **14**(4): 375-80.
115. Tigani X, Artemiadis AK, Alexopoulos EC, Chrousos GP, Darviri C. Self-rated health in centenarians: a nation-wide cross-sectional Greek study. *Arch Gerontol Geriatr* 2012; **54**(3): e342-8.
116. Enroth L, Veenstra M, Aartsen M, Kjaer AA, Nilsson CJ, Fors S. Are there educational disparities in health and functioning among the oldest old? Evidence from the Nordic countries. *European Journal of Ageing* 2019; **16**(4): 415-24.
117. Grundy E, Jitlal M. Socio-demographic variations in moves to institutional care 1991–2001: a record linkage study from England and Wales. *Age and Ageing* 2007; **36**(4): 424-30.
118. Himes CL, Wagner GG, Wolf DA, Aykan H, Dougherty DD. Nursing home entry in Germany and the United States. *Journal of Cross-Cultural Gerontology* 2000; **15**(2): 99-118.
119. Lakdawalla DN, Schoeni R. Is nursing home demand affected by the decline in age difference between spouses? *Demographic Research* 2003; **8**(10): 279-304.
120. Martikainen P, Moustgaard H, Murphy M, et al. Gender, Living Arrangements, and Social Circumstances as Determinants of Entry Into and Exit From Long-Term Institutional Care at Older Ages: A 6-Year Follow-Up Study of Older Finns. *The Gerontologist* 2009; **49**(1): 34-45.
121. McCann M, Grundy E, O'Reilly D. Why is housing tenure associated with a lower risk of admission to a nursing or residential home? Wealth, health and the incentive to keep ‘my home’. *Journal of Epidemiology and Community Health* 2011.
122. Shea D, Davey A, Femia EE, et al. Exploring Assistance in Sweden and the United States. *The Gerontologist* 2003; **43**(5): 712-21.
123. Tomiak M, Berthelot J-M, Guimond E, Mustard CA. Factors Associated With Nursing-Home Entry for Elders in Manitoba, Canada. *The Journals of Gerontology: Series A* 2000; **55**(5): M279-M87.
124. Van den Bosch K, Geerts J, Willemé P. Long-term care use and socio-economic status in Belgium: a survival analysis using health care insurance data. *Archives of Public Health* 2013; **71**(1): 1.
125. Roe-Prior P. Sociodemographic variables predicting poor post-discharge outcomes for hospitalized elders with heart failure. *Medsurg nursing : official journal of the Academy of Medical-Surgical Nurses* 2007; **16**(5): 317-21.
126. Maniecka-Bryła I, Drygas W, Bryła M, Dziankowska-Zaborszczyk E. Determinants of Self-Rated Health among the Elderly Living in a Big City Environment. *Polish Journal of Environmental Studies* 2011; **20**(3): 691-9.
127. Ament BH, de Vugt ME, Koomen FM, Jansen MW, Verhey FR, Kempen GI. Resources as a protective factor for negative outcomes of frailty in elderly people. *Gerontology* 2012; **58**(5): 391-7.
128. Breeze E, Fletcher AE, Leon DA, Marmot MG, Clarke RJ, Shipley MJ. Do socioeconomic disadvantages persist into old age? Self-reported morbidity in a 29-year follow-up of the Whitehall Study. *American Journal of Public Health* 2001; **91**(2): 277-83.
129. Kiuila O, Mieszkowski P. The effects of income, education and age on health. *Health Econ* 2007; **16**(8): 781-98.
130. Law MR, Cheng L, Worthington H, et al. Impact of income-based deductibles on drug use and health care utilization among older adults. *Canadian Medical Association Journal* 2017; **189**(19): E690-E6.
131. Lee M, Khan MM, Brandt HM, Salloum RG, Chen B. Decomposing socioeconomic disparities in the use of colonoscopy among the insured elderly population before and after the Affordable Care Act. *Cancer Causes & Control* 2020; **31**(11): 1039-48.
132. Lima-Costa MF, Steptoe A, Cesar CC, De Oliveira C, Proietti FA, Marmot M. The influence of socioeconomic status on the predictive power of self-rated health for 6-year mortality in English and Brazilian older adults: the ELSA and Bambui cohort studies. *Ann Epidemiol* 2012; **22**(9): 644-8.

133. Low G, Keating N, Gao ZW. The Differential Importance of Personal and Environmental Resources to Older Canadians. *Canadian Review of Sociology-Revue Canadienne De Sociologie* 2009; **46**(4): 371-92.
134. Ornstein KA, Garrido MM, Bollens-Lund E, et al. The Association Between Income and Incident Homebound Status Among Older Medicare Beneficiaries. *Journal of the American Geriatrics Society* 2020; **68**(11): 2594-601.
135. Huijts T, Eikemo TA, Skalicka V. Income-related health inequalities in the Nordic countries: examining the role of education, occupational class, and age. *Soc Sci Med*; **71**(11): 1964-72.
136. Lucchetti M, Corsonello A, Fabbietti P, et al. Relationship between socio-economic features and health status in elderly hospitalized patients. *Arch Gerontol Geriatr* 2009; **49**: 163-72.
137. Merlo J, Gerdtham UG, Lynch J, Beckman A, Norlund A, Lithman T. Social inequalities in health- do they diminish with age? Revisiting the question in Sweden 1999. *Int J Equity Health*; **2**(1): 2.
138. Siciliani L, Verzulli R. Waiting times and socioeconomic status among elderly Europeans: evidence from SHARE. *Health Econ*; **18**(11): 1295-306.
